# Dynamic thresholding and tissue dissociation optimization for CITE-seq identifies differential surface protein abundance in metastatic melanoma

**DOI:** 10.1038/s42003-023-05182-6

**Published:** 2023-08-10

**Authors:** Ulrike Lischetti, Aizhan Tastanova, Franziska Singer, Linda Grob, Matteo Carrara, Phil F. Cheng, Julia M. Martínez Gómez, Federica Sella, Veronika Haunerdinger, Christian Beisel, Mitchell P. Levesque

**Affiliations:** 1 Department of Biosystems Science and Engineering, ETH Zurich, Mattenstrasse 26, 4058 Basel, Switzerland; 2 Department of Dermatology, University Hospital Zurich, University of Zurich, Zurich, Switzerland; 3 ETH Zurich, NEXUS Personalized Health Technologies, Wagistrasse 18, 8952 Schlieren, Switzerland; 4 SIB Swiss Institute of Bioinformatics, Zurich, Switzerland; 5 Present Address: Department of Biomedicine, University Hospital Basel, University of Basel, 4031 Basel, Switzerland

**Keywords:** RNA sequencing, Gene expression analysis

## Abstract

Multi-omics profiling by CITE-seq bridges the RNA-protein gap in single-cell analysis but has been largely applied to liquid biopsies. Applying CITE-seq to clinically relevant solid biopsies to characterize healthy tissue and the tumor microenvironment is an essential next step in single-cell translational studies. In this study, gating of cell populations based on their transcriptome signatures for use in cell type-specific ridge plots allowed identification of positive antibody signals and setting of manual thresholds. Next, we compare five skin dissociation protocols by taking into account dissociation efficiency, captured cell type heterogeneity and recovered surface proteome. To assess the effect of enzymatic digestion on transcriptome and epitope expression in immune cell populations, we analyze peripheral blood mononuclear cells (PBMCs) with and without dissociation. To further assess the RNA-protein gap, RNA-protein we perform codetection and correlation analyses on thresholded protein values. Finally, in a proof-of-concept study, using protein abundance analysis on selected surface markers in a cohort of healthy skin, primary, and metastatic melanoma we identify CD56 surface marker expression on metastatic melanoma cells, which was further confirmed by multiplex immunohistochemistry. This work provides practical guidelines for processing and analysis of clinically relevant solid tissue biopsies for biomarker discovery.

## Abstract (web summary)

CITE-seq analysis in healthy skin, and both primary vs. metastatic melanoma samples suggests practical considerations of different skin dissociation protocols and dynamic thresholding of antibody signals for biomarker discovery.

## Introduction

The last decade has been marked by immense progress in the field of single-cell RNA sequencing (scRNA-seq)^1,2^. As an unbiased technology that does not profile a panel of preselected transcripts, it is ideally suited to resolve cellular heterogeneity in complex healthy or diseased tissues. Single-nuclei RNA-sequencing has been proposed for larger cohorts of fresh-frozen samples or where tissue dissociation is hard to achieve, such as for mature interconnected brain tissues^3–5^ but this is not without its drawbacks^6^. Notably, both single-cell and single-nuclei RNA-seq do not address the gap between RNA and protein expression, which can result from technical capture difficulties (lack in transcript capture/drop-out) and biological processes (translational impediments, post-translational influences, RNA degradation kinetics or protein trafficking to and from the cell surface)^7,8^. Compared to other single-cell proteomics technologies such as flow and mass cytometry, cellular indexing of transcriptomes and epitopes by sequencing (CITE-seq) allows simultaneous capture of gene and protein information as well as the implementation of larger antibody panels by virtue of a highly diverse combinatorial DNA sequence space that can be utilized to tag individual antibodies, thereby avoiding previous limitations of spectral overlap or matching isotope selections^9^. Increasing antibody panel size is essential for improved cell type and cell state resolution of highly heterogeneous tissue samples such as clinical specimens as well as for novel biomarker discovery. The same principle of DNA-barcoded antibodies can be applied for sample multiplexing by cell hashing through the use of antibodies targeting ubiquitously expressed epitopes, which reduces experimental costs, allows doublet detection, and minimizes batch effects^10^.

Despite their differences in signal detection, CITE-seq, and flow cytometry share the same requirements of careful experimental setups and considerations in panel design. This includes testing antibody panels for detection of the epitopes of interest, working with optimal antibody titers, washing out unbound antibodies after staining, including appropriate controls, and addressing background staining signals^11^. However, tailoring an antibody panel is easier for biological samples where the cell type composition is known or expected a priori, such as for PBMCs or healthy tissues. In disease states such as cancer, heterogeneous sample types can vary substantially in tumor content and tumor microenvironment composition from, e.g., low to high tumor presence and immune-rich to stroma-rich sample types. Moreover, clinical biopsies are often limited in tissue size, making antibody panel testings practically difficult.

Currently, CITE-seq applications focus mainly on immunophenotyping of liquid^12–14^ and solid biopsies^15,16^. Opening the field to clinically relevant solid tissue samples comes with the challenge of potential bias in gene expression (GEX)^17–19^ and surface protein expression (SPEX)^20,21^ introduced by dissociation and cell handling processes. Most tissue types require enzymatic or mechanical processing steps. The proteolytic enzymes used to dissociate the extracellular matrix and cell–cell-junctions require incubation at 37 °C for a prolonged time for optimal function, which can cause stress responses in cellular transcriptomes^17–19^. Hence, whenever the tissue sample allows, cold digestion or mechanical dissociation is preferred to avoid these stress signatures^22,23^. In addition, the selection of enzyme cocktails, incubation times, and steps will also influence the types of released cells, with specific cell types being either more sensitive to the dissociation process or requiring extended dissociation to be released from the tissue^24^. Lastly, the proteolytic activity of digestion enzymes can cleave cell surface proteins, which poses a problem for surface protein measurement techniques such as cytometry and CITE-seq. Trypsin and dispase are examples of such enzymes^25^. In summary, the choice of tissue dissociation protocol can greatly affect the observed cell type composition, gene expression, and the spectrum of detectable surface proteins making protocol optimization to minimize these effects imperative. This could be in the form of optimizing for shortest and least harsh dissociation protocols that result in high cell release, avoiding surface protein-cleaving enzymes such as trypsin, and testing if the obtained cell composition matches expected ratios.

Protein expression is usually inferred from RNA expression and vice versa. However, while protein concentrations positively correlate with transcript abundances, the association is not strong^7,26,27^. The correlation coefficient of RNA and protein expression has been reported to be between 0.5 and 0.7. Large differences could be seen depending on whether observations are based on single-cell or population level^7^, with lower correlations on cell level attributed to the noise observed in scRNA-seq data, such as technical dropouts or differences in biological half-lives between RNA and protein^28^. RNA-protein correlations are also dependent on measuring technologies and their precision and accuracy in feature (RNA or protein) detection as well as on types of data analyses^29,30^. For example, correlations vary depending on whether they are calculated between or within features. Between-feature correlations take the average of all feature correlations per tissue leading to higher correlations, while within-feature correlations are proposed to be more appropriate as they take feature-specific variation in RNA-protein correlations into account^30^. Thus, with low protein predictions from RNA levels for many features, simultaneous profiling of both is necessary to identify actionable targets, although this is rarely performed^31,32^.

Here, we report the performance of CITE-seq on a range of liquid and solid tissue biopsies. We developed an improved bioinformatics technique using cell type-specific ridge plots on which we implemented antibody signal thresholds to account for background staining signals and visualized their variance across experiments. Solid biopsies included healthy skin, for which we established an optimized dissociation protocol and primary and metastatic melanoma samples. Using a PBMC model, we assessed dissociation-specific gene perturbations and epitope loss on immune cell populations. We evaluated RNA-protein codetection and calculated feature-specific correlations on aggregated sample and single-cell level and report varying correlations depending on the profiled feature, the cohort, and the type of analysis (sample or single cell-based). Finally, we demonstrated the potential for biomarker discovery by applying large antibody panels with minimal prior selection of included surface markers. In summary, we highlight the applicability and possible pitfalls of CITE-seq for liquid and solid tissue samples and paired differential gene expression and protein abundance analysis on clinical samples.

## Results

### Workflow overview

**Fig. 1:**
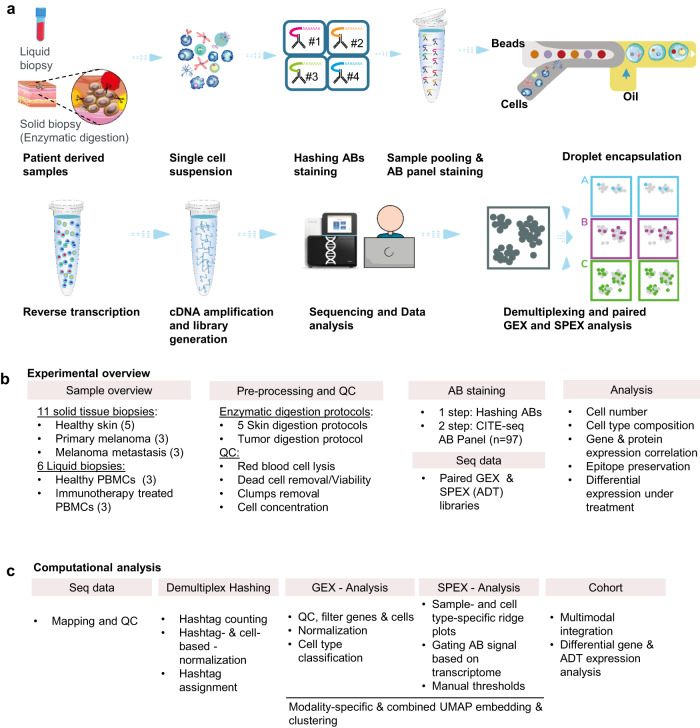
Study overview. **a** Workflow of sample processing. **b** Experimental overview. **c** Computational analysis. AB Antibody, ADT antibody derived tag, QC Quality control.

CITE-seq and cell hashing were performed on liquid and solid tissue biopsies (Fig. 1a, b). Experimentally, cells from 17 samples (Supplementary Data 2) were hashed and stained with a panel of 97 antibodies (Supplementary Data 2) covering key as well as exploratory immuno-oncology markers resulting in 57,261 cells after preprocessing and quality control. Biopsies included slow-frozen biobanked PBMCs, healthy skin, primary melanoma, and lymph node (LN) melanoma metastasis samples. For skin biopsies, five different dissociation protocols were compared for optimal yield, viability, and epitope preservation (Supplementary Note 1). Stringent red blood cell lysis, dead cell, and clump removal improved the quality of the final single-cell suspension prior to CITE-seq processing. Computationally, we implemented cell type-specific gating and setting of sample-specific manual thresholds in analogy to FACS analysis allowing us to perform RNA-protein correlation and differential abundance (DA) analysis (Fig. 1c). Finally, experimental and analytical improvements demonstrated CITE-seq utility for multimodal single-cell profiling and its potential for biomarker discovery.

### Dynamic thresholding for the removal of background noise improves cell surface protein signal detection

Taking advantage of single-cell resolution and gene expression-based cell type annotation, we first implemented major cell type-specific protein abundance ridge plots for each sample’s SPEX analysis to identify the protein feature abundance. This strategy is visualized on healthy PBMCs (Supplementary Fig. 1a). In contrast to all-cells protein abundance distribution plots where a marker is plotted for all cells within the sample (Supplementary Fig. 1b), protein abundance visualized on cell type-specific ridge plots allowed for marker detection on individual cell populations (Supplementary Fig. 1c). Furthermore, manual thresholds were set to reduce background noise and increase confidence in antibody signal detection (Supplementary Data 1, Supplementary Data 2) as well as to capture positive antibody signals from cell populations present at a lower frequency (Supplementary Fig. 1). This approach allowed to confirm protein detection on expected cell types as for CD2 on lymphocytes and rare cell types such as CD16 on natural killer (NK) cells or CD123 on plasmacytoid dendritic cells (Supplementary Fig. 1d and e). Finally, we visualized threshold variation for the top 30 antibodies with the largest variance across experiments underscoring that thresholds vary across experiments and cannot be easily automated or transferred from one experiment to another (Supplementary Fig. 2, Supplementary Data 1). In summary, manual thresholds need to be adapted for each experiment and processed samples, but allow for background signal removal in CITE-seq antibody analysis.

Because of the improved antibody signal detection, we applied the manual thresholds on all samples throughout this study.

### Optimization of healthy skin dissociation protocols

**Fig. 2:**
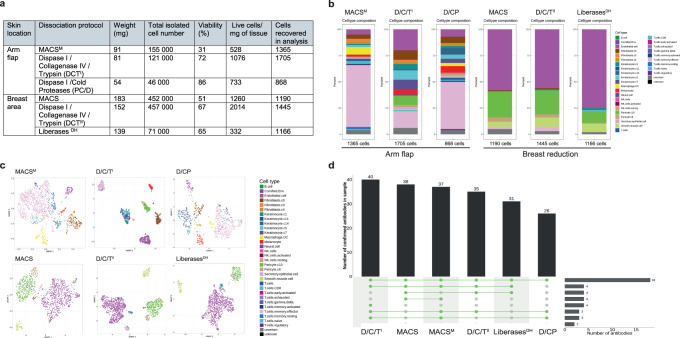
Protocol optimization for healthy skin dissociation. **a** Table summarizing dissociation efficiency of tested protocols (*n* = 2, biologically independent samples). **b** Cell type composition bar plots, and **c** UMAP plots, of each tested protocol and biopsy type. **d** UpSet plot showing number of detected antibodies per tested protocol.

**Fig. 3:**
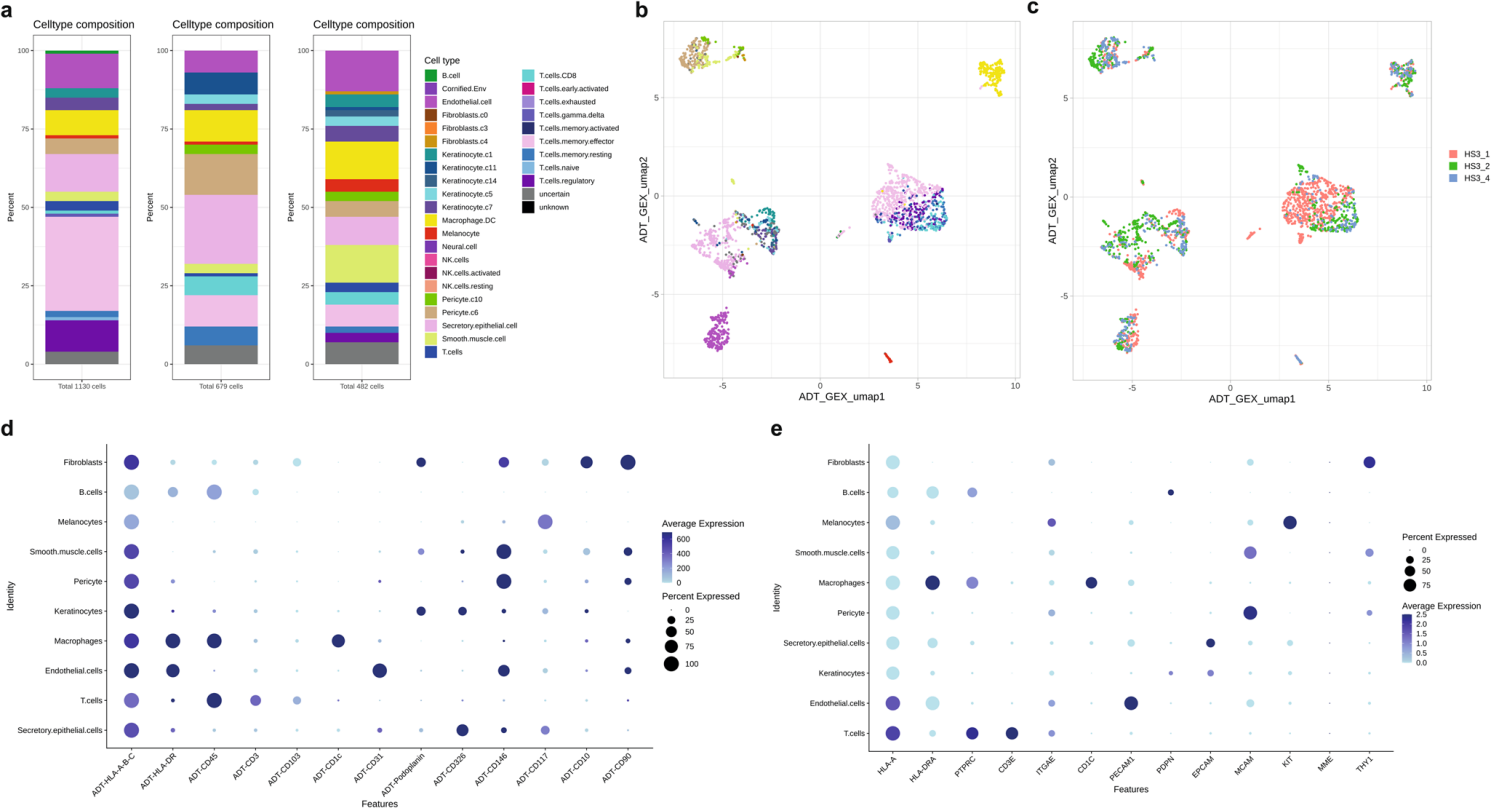
CITE-seq performance on healthy skin biopsies. **a** Cell type composition bar plots per sample; left, HS3_1; middle, HS3_2; right, HS3_4 (*n* = 3, biologically independent samples). **b** UMAP plots with cell type, and **c** sample information. **d** Protein and **e** gene lineage marker expression dot plots per major cell type.

The main objective of the study was to establish CITE-seq for skin and primary cutaneous melanoma samples. Skin is a collagen-rich tissue that is difficult to dissociate, requiring several enzymatic digestion steps^33^. Therefore, we set out to optimize a dissociation protocol suitable for CITE-seq applications. The following five protocols were tested on surplus healthy skin material from surgery (arm and breast areas, Fig. 2, Supplementary Data 2): MACS skin dissociation kit with and without mechanical dissociation using the gentleMACS Octo Dissociator (MACS^M^ and MACS, respectively), three-step tissue dissociation protocols consisting of consecutive Dispase I, Collagenase IV and Trypsin with EDTA (0.25%) (D/C/T), a combination of Dispase I and cold-active protease (D/CP) and Liberases^DH^. In both experiments, the D/C/T protocol had the highest dissociation efficiency, defined as the number of isolated viable cells per mg of tissue with 2–6 fold more released cells (Fig. 2a, Supplementary Data 2). Moreover, this protocol yielded the highest number of cells in the analysis and was able to capture a heterogeneous cell type composition including keratinocytes, melanocytes (in the UV-exposed arm flap skin sample), fibroblasts, and immune cells (Fig. 2b, Supplementary Data 1) with clear cell type clustering (Fig. 2c). In comparison, the MACS protocol-treated sample did not capture the pericyte population; however, it contained a high number of T cells compared to D/C/T and D/CP. Furthermore, the D/C/T protocol yielded high numbers of detected antibodies with 40 and 35 antibodies confirmed in arm flap and breast tissues, respectively (Fig. 2d, Supplementary Data 1, Supplementary Data 2), indicating it retained many epitopes. The selected D/C/T (SkinD) protocol was then applied to a cohort of three healthy skin biopsies from the head and neck area (Fig. 3). The three samples shared similarly diverse cell type compositions including keratinocytes, melanocytes, pericytes, immune cells, and endothelial cells (Fig. 3a, Supplementary Data 1). Combined sample UMAP visualization showed clear clustering into different cell types (Fig. 3b), while the clustering was not affected or biased by hashing or sample origin (Fig. 3c). Finally, cell lineage protein (Fig. 3d, Supplementary Data 1) and gene (Fig. 3e, Supplementary Data 1) marker expression confirmed the cell identities.

### A PBMC model reveals the effects of enzymatic treatment on gene expression and epitope preservation

**Fig. 4:**
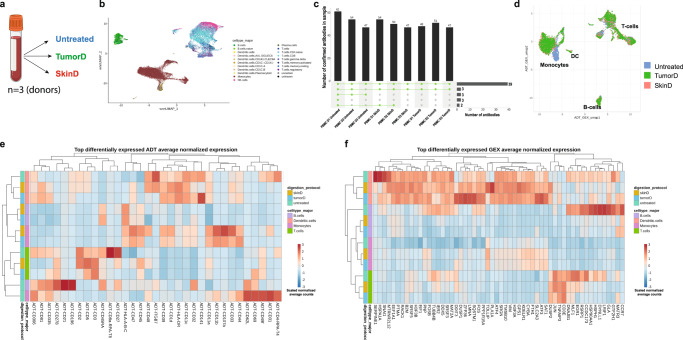
PBMC model revealing the influence of enzymatic dissociation on gene expression and epitope preservation. **a** Experiment design: healthy PBMC from three healthy donors were aliquoted each in three parts that were left untreated at 37 °C for 1 h or underwent SkinD or TumorD enzymatic incubation at 37 °C for 1 h. **b** Integrated UMAP visualization of all 9 samples (3 donors, 3 treatment conditions) with cell type information. **c** UpSet plot showing confirmed antibody detection across all samples. **d** Representative UMAP plot from Donor 3 showing protocol-specific influence on clustering of different cell types. **e** Differential SPEX and **f** GEX heatmap by sample and major cell type aggregated over three donors showing top differentially expressed features across treatment conditions.

CITE-seq is mainly used on liquid biopsies, which do not require extensive tissue dissociation steps that could affect cell surface protein presentation. In order to investigate the impact of enzymatic treatments on gene expression and epitope preservation on major immune cell populations, we tested the optimized skin dissociation protocol (SkinD, D/C/T) and a well working solid soft tumor dissociation (TumorD, Supplementary Note 1) protocol^34^ on PBMC aliquots of three healthy donors against an untreated control (Fig. 4a). Individually treated aliquots of each patient were hashtag-antibody stained, washed and pooled, followed by staining with the panel comprising 97 antibodies (Supplementary Data 2). Stained pools were washed and processed for single-cell sequencing. UMAP visualization of all samples (three donors, each with three treatment conditions) showed robust clustering into B cell, T cell, and myeloid/monocyte populations (Fig. 4b). Major cell types from each donor and treatment condition were pooled and selected protein marker expression was plotted on non-thresholded and thresholded protein data, revealing the degree of background signal in the data and highlighting the impact of setting manual thresholds (Supplementary Fig. 3a and b), see also Grob et al.^35^. Next, antibody signal detection was assessed between different treatment conditions. Out of 97 tested antibodies, 39 were detected across all samples (Fig. 4c, Supplementary Data 1, Supplementary Data 2); untreated PBMCs showed the highest number with up to 61 detected antibody markers. In addition, three antibodies were detected in all conditions, except for the Donor 2 untreated sample (Fig. 4c, Supplementary Data 1, Supplementary Data 2). Looking at treatment-specific effects in individual samples, the monocyte population clustered separately from untreated ones in both dissociation protocols, while B and T cell populations were mainly unaffected (Fig. 4d, Supplementary Fig. 3c and d). To identify digestion-sensitive surface proteins on major cell types, we performed protein differential abundance (DA) analysis based on normalized counts using the Seurat function findMarkers. Loss of surface protein expression was observed under both digestion protocols in all cell types (Fig. 4e). Specifically, we observed loss of CD4, CD8, CD27, CD335 on T cells, CD335, CD31, CD49f, CD62L, CD69 on monocytes, CD21, CD81, CD196, CD278, CD335, CD336 on B cells as well as ITGB7, CD48, CD31, CD49f, and CD141 on dendritic cells. CD4 and CD336 loss was observed on T cells, dendritic cells, and monocytes, while CD45RA expression was decreased on both B and T cells (Fig. 4e). Moreover, the DA analysis between treatment conditions by cell type showed upregulation of various surface proteins (Fig. 4e). T cells showed an increase in CD3, CD5, and CD69 under either one or both of the digestion protocols. Cell surface proteins associated with antigen presentation (CD1d, HLA-A-B-C), adhesion and migration (CD11a, CD11b, CD44, CD47), immune regulation (CD39, CD107a) as well as activation (CD11c, CD32, CD33, CD48) were upregulated on monocytes (Fig. 4e). Differential gene expression (DE) between conditions showed upregulation of signatures of innate immune activation (*SLC2A3, TOLLIP, SAT1, FOSB)*, autophagy (*SQSTM1*), stress response and apoptosis (*DDIT3*, *DUSP1*) as well as downregulation of genes involved in heat shock response (*HSP1A1*, *HSP90AA1, HSPH1, DNAJB1*) in enzymatically treated cells compared to untreated (Fig. 4f). Thus, the PBMC model comparing untreated versus enzymatically treated conditions informed on dissociation-sensitive genes and identified not only loss of certain surface epitopes but also increased protein presentation by the enzymatic digestion.

### RNA and surface protein codetection

**Fig. 5:**
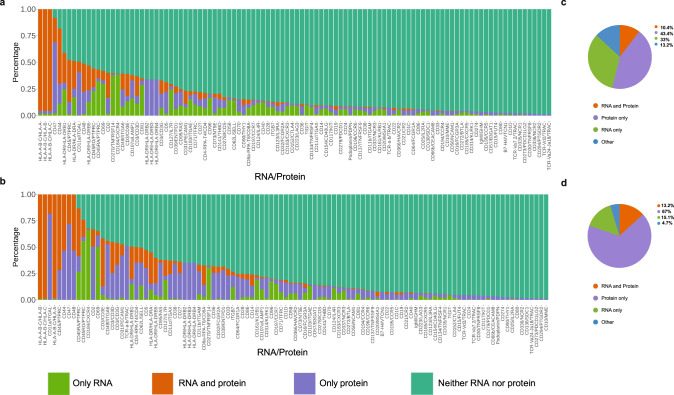
RNA and protein codetection plots. **a** Feature codetection waterfall plot of solid tissue biopsy cohort including five skin, three primary, and three metastatic melanoma samples (*n* = 11, biologically independent samples) and of **b** liquid biopsy cohort including PBMCs from three healthy donors (no digestion) and three immunotherapy-treated melanoma patients samples (*n* = 6, biologically independent samples). Plots show the percentages of cells with “RNA only”, “RNA and protein”, “protein only”, or “neither RNA nor protein” detection category. **c** Percentage of feature pairs in the solid and **d** liquid cohort that were predominantly assigned to one category based on a threshold of >50% of cells. Feature pairs where the threshold was not reached for either of the three categories were assigned to “other”.

Most differential mRNA expression studies assume the expression of the corresponding protein product, and some studies provide parallel protein stainings from paired formalin-fixed paraffin-embedded (FFPE) slides to demonstrate the translation of mRNA transcript into a protein product. Here, we assessed the codetection of RNA-protein pairs for the CITE-seq antibody panel (*n* = 97) in PBMCs from three healthy donors and three immunotherapy-treated melanoma patient samples (liquid biopsies, *n* = 6, no digestion applied) as well as in five healthy skin, three primary melanoma, and three lymph node metastatic melanoma samples (solid biopsies, *n* = 11, Supplementary Fig. 4, Supplementary Data 2). We analyzed codetection in RNA-protein detection in each cell of the solid and liquid biopsy cohorts by looking at four conditions (Fig. 5a and b, respectively; RNA-protein pairs sorted by decreasing codetection, Supplementary Data 1. Supplementary Fig. 4; alphabetically listed RNA-protein pairs): (i) detection of RNA only (minimal count of 1 transcript per cell), (ii) detection of protein only (signal above manually defined threshold per cell; Supplementary Fig. 1, Supplementary Data 1), (iii) detection of both RNA and protein, and (iv) absence of both RNA and protein. Next, for both solid and liquid biopsy cohorts, we assessed to which category (“RNA only”, “protein only”, “RNA and protein”) each feature was predominantly assigned (>50% of cells per category) and calculated the percentages of these categories by cohort (Fig. 5c, d, Supplementary Data 1). The first 15 markers that showed the highest codetection in “RNA and protein” expressions were shared between solid and liquid biopsies (left side of the waterfall plots, Fig. 5a, b, Supplementary Data 1). We found that with 43.4% and 67.0%, most of the markers were predominantly detected only at the protein level, while 33.0% and 15.1% markers were detected at the RNA level only, and to the lowest extent with 10.4% and 13.2% markers were detected on RNA and protein levels in solid and liquid biopsy cohorts, respectively (Fig. 5c, d, Supplementary Data 1). “Protein only” and paired “RNA and protein” were detected more often in liquid biopsies compared to solid biopsies. In both biopsy cohorts, overlap in RNA and protein detection was observed in cell surface markers important for regular cell functions such as major histocompatibility antigens (HLA-A/B/C, HLA-DR), cell lineage markers (CD45, CD3, CD8a), cell surface glycoproteins (CD44) and immunoglobulins (CD48). CD45RO and CD45RA are isoforms used to distinguish between different naïve and memory subpopulations, which could not be distinguished at the RNA level (*PTPRC*) without full-length transcript information. Higher CD45RA expression was observed in the liquid biopsy cohort, as expected in peripheral blood with a higher fraction of naïve cells^36^; whereas, in solid biopsies, CD45RA and CD45RO were detected on the same level indicating that immune cells found in the tissues are more activated and/or differentiated. Markers that showed “RNA only” expression included chemokine receptors and activation markers such as *CXCR3* (CD183), *CXCR4* (CD184), *IL4R* (CD124), *IL7R* (CD137), *CCR7* (CD197), *LAG3* (CD223), and *TIGIT*. Markers predominantly detected at the “protein only” level included among others CD10, CD39, CD36, CD56, B7-H4, CD195, and CD70 with missing GEX information indicating low RNA expression or difficulties in transcript capture. In both liquid and solid biopsy samples, “protein only” expression was detected in cases where no single RNA directly translates into the epitope such as various types of T cell receptor epitopes (TCR-α/β, TCRV δ2, TCR-Vα7.2, TCR-Vα24-Jα18) and CD57 (glycoepitope). In the case of the B cell immunoglobulin M (IgM) isotype, the immunoglobulin heavy constant mu gene (*IGHM*) could not be detected because the gene is located on the 3-prime end of the B cell receptor mRNA molecule^37^, which was not covered with our 5-prime sequencing approach. The decreased protein detection in the solid cohort, seen as a higher fraction of “RNA only” detection, was attributed to enzymatic digestion-associated loss of epitopes as shown in the PBMC model (Fig. 4e). In summary, the assessment of RNA-protein codetection highlighted similarities and differences in feature detection in liquid and solid biopsy cohorts.

### RNA and protein expression correlation

**Fig. 6:**
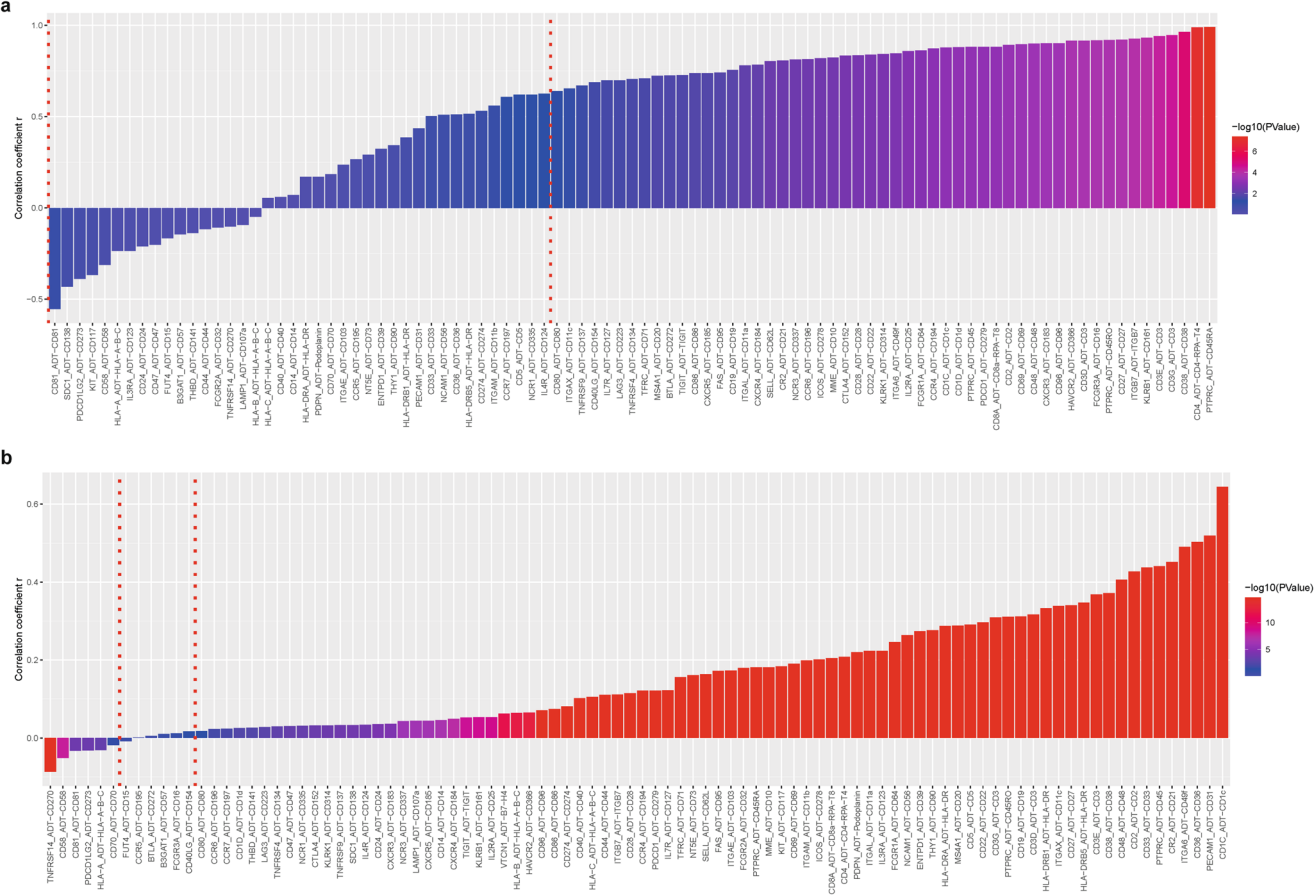
RNA and protein expression correlation. Pearson correlation coefficients of 93 RNA-protein pairs on aggregated **a** sample level (*n* = 11, biologically independent samples) and **b** cell level (*n* = 12,665 single cells) for the solid tissue biopsy cohort. The Pearson correlation coefficient between each RNA-protein pair is shown along with its related *p*-value. The significance threshold is set to 0.05 and indicated as a red, dotted vertical line. Pairs were excluded if either one or both members of the pair were not detected.

Next, we asked how well RNA and protein expression is correlated and calculated RNA-protein correlation coefficients on a sample level (Fig. 6a, Supplementary Fig. 5a, Supplementary Data 1) and a cell level (Fig. 6b, Supplementary Fig. 5b, Supplementary Data 1) for the solid biopsy (Fig. 6, Supplementary Data 1) and the liquid biopsy cohorts (Supplementary Fig. 5). Only RNA-protein pairs were included for which both features were detected. More RNA-protein pairs with significant positive correlations (*p* < 0.05) were detected in the solid cohort compared to the liquid cohort on a sample (57% vs. 25%) and cell (88% vs. 80%) level reflecting broader feature detection due to a higher cell type diversity. In addition, the higher number of solid samples provided better statistical power compared to the liquid biopsy cohort. Strong differences were observed depending on whether correlations were computed on a sample (population) or single-cell level. While only 57% (53 out of 93 included pairs) of RNA-protein pairs reached positive significance on a sample level in the solid cohort, the correlation of these was high (mean *r* = 0.83). On a single-cell level, 88% (82 out of 93 pairs) of pairs were significantly positively correlated in the solid cohort; however, these displayed an overall lower correlation (mean *r* = 0.17). Only three features (3% of all pairs) showed a correlation coefficient above 0.5 and six pairs (6% of all pairs) were significantly anti-correlated, albeit with coefficients equal to or lower than 0.09, indicating that noise or other small fluctuations can reach significance with a large number of observations. The same was observed for significantly positively correlated pairs in the liquid cohort with mean *r* = 0.92 on a sample and mean *r* = 0.24 on a cell level. In summary, this analysis provides a list of highly correlated features that could guide antibody or gene panel design in future studies.

### RNA and protein abundance analysis of PBMCs

**Fig. 7:**
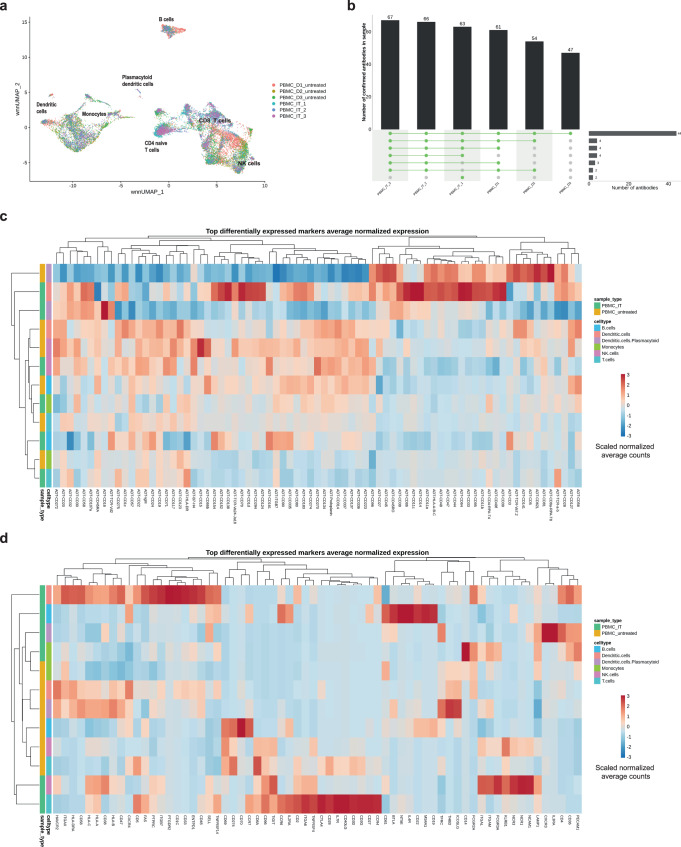
Differential protein abundance analysis in PBMCs from healthy donors and immunotherapy-treated melanoma patients. **a** UMAP visualization of 6 samples by sample identifier with labeled cell types (healthy donors *n* = 3, immunotherapy treated melanoma patients *n* = 3, biologically independent samples). **b** Antibody detection per sample. **c** Differential protein abundance and **d** differential gene expression analysis by treatment and cell type.

Application of CITE-seq to DA and DE analysis was assessed on a PBMC cohort consisting of three healthy donors and three immunotherapy-treated (IT) melanoma patients (Supplementary Data 2). UMAP visualization showed cell clustering according to distinct cell types, and not according to sample origin (Fig. 7a). IT-treated patients’ PBMCs showed higher numbers of detected antibodies, which included CD161, CD137, CD272, and CD335 markers (Fig. 7b, Supplementary Data 1, Supplementary Data 2). We next performed DA/DE analysis between PBMCs from healthy donors and IT-treated melanoma patients on SPEX (Fig. 7c) and GEX (Fig. 7d) levels, respectively. Because the thresholding introduces changes in count distribution, DA was computed on non-thresholded data, however, thresholds were applied in the final results of the visualization of the heatmaps (see methods). DE analysis on GEX level detected strong upregulation of activation, checkpoint, and memory markers such as *CTLA4, TIGIT*, and *CD27* on IT-treated T cells, a finding that could not be verified on SPEX level due to above reported poor protein detection of these markers. The absence of CTLA-4 protein expression could be explained by anti-CTLA-4 treatment in case the epitope is already occupied by a therapeutic antibody. However, one of the IT-treated patients received anti-PD-L1 treatment only (Supplementary Data 2). Therefore, to validate the CITE-seq results, the same infusion of PBMCs from three patients that were processed for CITE-seq were selected for an orthogonal experiment. To test variability in antibody clone performance, in addition to the clone included in the CITE-seq panel (BNI3), clone L3D10 was selected based on reports from other studies^38^. Interestingly, although CTLA-4 is a surface receptor, both instructions from the manufacturer and previous publications on CTLA-4 expression on T-cells^39^ utilize prior cell stimulation and intracellular staining to detect the protein expression. Therefore, PBMCs were first stimulated for 4 hours with PMA/ionomycin, followed by extracellular and intracellular staining. Cells were gated as lymphocytes/single cells/CD3+/CD4 + CD8− and gates for CTLA-4 were adjusted according to the matching isotype control (Supplementary Fig. 6a).CTLA-4 protein expression was only observed when both stimulation and intracellular staining was performed (Supplementary Fig. 6b and c). In addition, compared to the L3D10 clone, lower CTLA-4 detection was observed using the BNI3 clone (Supplementary Fig. 6b and c).

Overall, GEX and SPEX DE analysis provided different results as a consequence of feature limitations in SPEX space and different capture strengths of both types of measured molecules.

### Proof-of-concept CITE-seq application on a melanoma cohort for biomarker discovery: differential protein abundance and validation

**Fig. 8:**
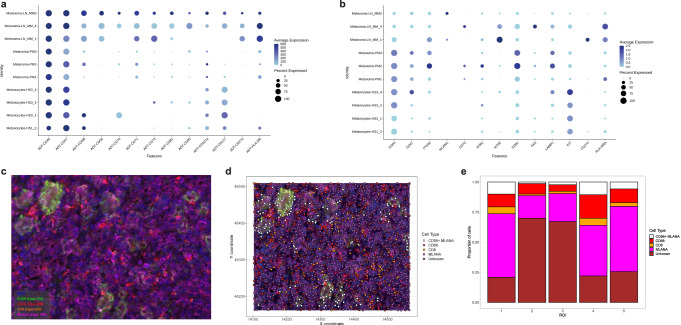
Proof of concept CITE-seq application for biomarker discovery on a cohort of solid tissues. **a** Protein and **b** gene marker expression dot plot per cell type and sample. Melanocytes from healthy skin samples (*n* = 4, biologically independent samples, dissociated with D/C/T protocol—the skin biopsy from breast reduction was excluded from this analysis because of lack of melanocyte presence), primary melanoma cells from primary melanoma samples (*n* = 3, biologically independent samples), and metastatic melanoma cells from the metastatic lymph node samples (*n* = 3, biologically independent samples). **c** Multiplex immunohistochemistry staining of a matched metastatic melanoma lymph node tissue sample showing CD56 signal on MLANA-positive melanoma cells. **d** Analysis of a representative region of interest (ROI) quantifying single cells positive for: CD8 (T cells), CD68 (macrophages), MLANA (melanoma cells), and DAPI (nuclei staining). **e** Cell type composition bar plots from five analyzed ROIs.

As a proof-of-concept study of CITE-seq applicability for biomarker discovery, we next performed differential protein abundance analysis on healthy skin melanocytes, primary melanoma, and metastatic melanoma cells and visualized protein and corresponding RNA expression (Fig. 8a, b, Supplementary Data 1). Various markers associated with immune evasion (CD47 and CD274)^40,41^, invasion and metastasis initiation (CD44, CD49f, CD81, and HLA-DR)^42,43^ were found on metastatic melanoma cells. HLA-DR was detected at low, medium, and high levels in melanocytes, primary melanoma, and metastatic melanoma cells, respectively, although these biopsies contained BRAF and not the NRAS mutation, which is associated with higher HLA-DR expression^43,44^. CD107a abundance, a marker for organelles of endosomal-lysosomal lineage and proxy for vesicle secretion^45^, was detected on a larger fraction of healthy melanocytes and reduced on primary and metastatic melanoma cells (Fig. 8a, Supplementary Data 1, Supplementary Data 2). CD117 expression was detected on healthy melanocytes and metastatic melanoma cells but absent on primary melanoma cells. Metastatic melanoma cells showed CD81 expression, a marker associated with metastatic progression^46^ and high expression of CD49f. In addition, protein expression was observed for CD56 (neural cell adhesion molecule 1 (*NCAM1*), involved in cell–cell adhesion), CD71 (transferrin receptor 1)^47^ and CD73 (ecto-5'-nucleotidase responsible for extracellular adenosine production with immunosuppressive function)^48,49^. CD56 (*NCAM1*) as well as CD274 (*PD-L1*) were only detected in metastatic melanoma cells and primarily on a protein-only level (Fig. 8a, b) consistent with the overall detection pattern for these markers in liquid and solid cohorts (Fig. 5a, b, Supplementary Fig. 4). While CD274 (PD-L1) is a known immune checkpoint inhibitor^50,51^, less is reported on CD56 expression on metastatic melanoma cells^52^. To validate and spatially resolve the expression of CD56, a paired FFPE section of the metastatic melanoma LN was analyzed using Akoya multiplex immunohistochemistry (Fig. 8c). Quantification of CD56/MLANA double-positive melanoma cells, MLANA+ melanoma cells, CD68+ macrophages, and CD8+ T-cells in five regions of interest (ROI) (Fig. 8d, e, Supplementary Data 1) showed that on average 13% of all MLANA+ cells (3,171 cells) were CD56/MLANA double-positive (430 cells).

## Discussion

Application of antibody panels for multiplexed protein detection requires careful tailoring of optimal working concentrations, which is typically established by titration experiments. For assays in which antibodies are conjugated to fluorophores (flow cytometry) or isotopes (mass cytometry), compensation steps for each conjugate are performed to separate the true positive signal from the background staining. In addition, the number of antibodies in the panels is limited by the number of unique conjugates due to technological limitations to resolve the signals. Oligo-conjugated antibody barcoding technology such as CITE-seq is based on the unique DNA sequence and due to its immense combinatorial sequence space offers a virtually limitless number of antibodies to be included in the panel. When possible, reducing staining concentrations was proposed to decrease antibody-related sequencing costs^53^. Titration of a large antibody panel is costly, time-consuming, and not always possible when using clinical samples due to limited tissue material, which is a potential limitation when applying CITE-seq on patient-derived biopsies. In this study, we tested a panel of 97 antibodies without prior titration and achieved good signal-to-noise ratios allowing us to identify positive antibody signals. The background staining was resolved using transcriptome-based gating and plotting cell type-specific protein abundance ridge plots. First, the ridge plots visualize individual cell types, which allows antibody signal detection on rare cell populations that otherwise would have been lost on all-cells protein abundance distribution plots. Further, the background staining was removed by applying dynamic manual thresholds to display only true positive antibody signals. We also demonstrated that these thresholds vary between experiments and cannot be easily transferred from one dataset to another. With wider application of CITE-seq analysis, new approaches are being developed that aim to calculate the background staining from empty droplets as well as by simultaneous staining with non-human species directed antibodies^54,55^; however, manual examination of each protein marker would still be necessary to correct for possible inaccuracies.

Surface protein detection on solid tissues is more complicated due to the pre-processing into single-cell suspensions using mechanical or enzymatic digestion that introduces bias into cellular composition as well as affects the transcriptome and the surface proteome of the cells^56^. Dissociation of solid tissues using proteolytic enzymes is the most reliable method to obtain single-cell suspensions that capture the heterogeneity of the original tissue and optimization is required to obtain heterogeneous cell populations and preserve cell viability^25^. Immune cell release can be achieved by shorter treatment times (~15 min), while stromal cells require prolonged enzymatic treatment due to complex cell–cell and cell–matrix connections^57^. Although prolonged dissociation is often necessary to extract most cell types from tissues, it also introduces bias in gene expression by upregulating early stress^57^ and heat shock signatures^23^ and can cause artifactual loss of surface protein expression^21^. To investigate preservation of surface epitopes on immune cells, we treated healthy PBMCs with skin (SkinD) and tumor dissociation (TumorD) protocols and compared the RNA and protein expression patterns against an untreated control. In agreement with previous reports, enzymatically treated PBMCs showed loss of CD4, CD8, CD21, and CD62L in our CITE-seq analysis in samples treated by both SkinD and TumorD protocols^20,21^. CD141 signal was reduced in SkinD-treated dendritic cells, consistent with trypsin sensitivity as previously reported^58^. Interestingly, differential gene and surface protein expression analysis showed that lymphocytes were most affected by enzymatic digestion compared to other cell populations with loss of dissociation-sensitive epitopes (such as CD4, CD8, CD21). The monocyte population showed prominent gains in many cell surface markers such as CD107a (degranulation and activation), CD39 (metabolic activation), CD1d (antigen presentation) and CD33 (macrophage differentiation) indicating functional sensitivity to enzymatic exposure. This is in line with previous reports of transcriptomic and proteomic changes of microglia in enzymatically digested mouse brain tissues compared to mechanical dissociation^22^ and collagen-induced chemotaxis of human blood monocytes^59^.

Global single-cell transcriptome profiling of primary tissues from various tumors and non-malignant samples revealed intrinsic cell type heterogeneity and greatly improved our understanding of disease mechanisms and drug actions in multiple clinical fields^60–63^. However, scRNA-seq studies are limited only to the description of the transcriptome, while protein expression is either inferred from gene expression or orthogonally validated from paired FFPE samples^62^. Single-cell analysis pipelines are rapidly advancing into technologies that can integrate multiple profiling modalities, and recent progress in sequencing-based technologies allows simultaneous capture of cell surface protein and gene expression from a single cell^9,63,64^, thereby linking genes and their protein products and capturing a more realistic functional state. In clinical settings, CITE-seq was applied to monitor responses to therapy and relapse by Ibrutinib (irreversible Bruton Tyrosine Kinase inhibitor) treatment of chronic lymphocytic leukemia (CLL)^14^. The analysis revealed that CD3 is well identified by both protein-labeling as well as by gene expression, whereas CD69 or CD19 were mainly detected by the cell surface protein labeling. CITE-seq analysis showed a decrease in CD69 expression (prognostic factor) correlated to clinical progression in CLL at three months post-treatment, and subsequent increase at month 27 correlating with disease progression^14^. A study that applied CITE-seq to myeloid populations within human and mouse glioblastoma samples reported RNA-protein codetection for many markers; other features, however, were differentially expressed on protein level only^65^. In this study, we analyzed the detection intersection in 97 RNA-protein pairs, reported predominant detection (more than 50% of cells with at least one observation), and calculated their correlation. We corroborate previous observations of varying degrees of feature detection, provide calculations on feature correlation and list features in each category that can serve as a lookup table for future investigators. The high amount of features that could only be well detected in protein space highlights the additional value CITE-seq can provide considering clinically relevant readouts. Nevertheless, markers such as *TIGIT* and *LAG3* were better detected at the RNA level showing neither technology alone is superior in feature detection, thus highlighting the added value of combining both omics arms. Furthermore, transcript isoforms are not covered with the short read sequencing data. As noted by Liu et al.^29^, the possibility that protein levels may be compared to splice isoforms that do not contain the respective protein sequence may distort the RNA-protein correlation. An example of this is the detection of CD45RA and CD45RO splice isoforms of CD45 on immune cells, which could be detected with isoform-specific antibodies. Due to the lack of full-transcript sequence coverage, they could not be detected at the transcript level. Coupling long-read sequencing technologies allowing for isoform detection^66,67^ with surface protein capture would be the next logical step to further gain biological insights. More “protein only” and “RNA and protein” expression was observed in liquid biopsies (three healthy and three immunotherapy-treated PBMCs) or in solid biopsies that were digested with a milder enzymatic blend (metastatic melanoma in LN) indicating digestion-associated loss of cell surface proteins. The absence of positive antibody signals could also be influenced by other factors such as suboptimal antibody clone performance (e.g., CD4 and CD8 clones SK3 and SK8, performed better than clones RPA-T4 and RPA-T8, respectively). An additional limitation of the CITE-seq method might be due to how liquid biopsy is processed prior to single cell droplet generation. Usually, to have minimal impact on the transcriptome of cells derived from patients, samples are processed as quickly as possible. However, for certain surface proteins, such as CTLA-4, it has been described that surface receptors reside on the cell surface only for a short time, and are internalized in the absence of a ligand, which means that at any given time point, there is very low surface expression of CTLA-4^68^. In vitro stimulation is necessary for reliable detection of CTLA-4 expression, which is not feasible if fast sample processing is required. Therefore, surface protein expression of such markers might be overlooked during CITE-seq profiling. Moreover, our analysis compared well with RNA-protein detections in an independent study comprised of glioblastoma-isolated immune cells^65^, which showed higher protein detection for CD36, podoplanin, CD64, CD49f, CD86, CD15, CD56, CD90, CD11a and CD11b, high RNA and protein codetection for CD44 and higher RNA detection for *CXCR4* (CD184). This was consistent with our classifications of predominantly “protein only”, “RNA and protein”, and “RNA only”, indicating that technological and biological effects influencing RNA-protein detection are preserved across studies and experiments, thus supporting a wider application of CITE-seq in basic and translational research. CITE-seq applications on liquid biopsies enabled the identification of cell populations present at low frequencies, such as antigen-presenting cells (CD1c+ and CD141+ cells) or naïve and memory phenotypes by discriminating expression of CD45RA and CD45RO^69^. RNA and protein correlation analysis was calculated on a sample and cell level with more significantly positively correlated pairs found in the solid cohort due to higher cell diversity and sample numbers. Cell-based correlations provided more significantly correlated pairs resulting from a higher number of observations, however, had lower correlation coefficients. The generalized low correlation in single-cell observations reflected most likely differences in transcript detection, cell-to-cell variability, transcriptional noise, and potentially poor antibody binding and is in line with previously reported differences in correlations on sample (population) or single-cell levels^7^. This indicates that to mitigate these effects and to achieve higher confidence in feature detection, RNA-protein correlations should be investigated at least on a cluster or cell type instead of single-cell level.

Studies that used CITE-seq to analyze solid tissues so far have only focused on subtyping the immune milieu of various organs such as the brain^15,65^, kidney^70^, breast^16^, and skin^71^. Besides immune cells, the tumor microenvironment (TME) is composed of various cell types such as endothelial cells, pericytes, fibroblasts, and neural cells. As has been shown, the non-malignant components of the TME can also contribute to tumorigenesis, progression, and metastasis^72,73^. Therefore, understanding the cell–cell interactions as well as profiling the functional states of various cell types within the environment of the solid tissue samples is as important. Commonly, in single-cell protein profiling techniques such as flow cytometry or CyTOF, antibody panels are pre-selected and prioritized based on previous knowledge, and the combination of established and exploratory markers is technologically limited. For biomarker discovery in CITE-seq, in addition to well-established antibodies, we added a panel of exploratory markers and identified known as well as less described metastatic melanoma markers. After analysis of the panel, we compared the expression of the selected surface markers on healthy melanocytes, as well as primary and metastatic melanoma cells. Among markers identified on metastatic melanoma cells, protein expression did not correlate with the corresponding gene expression in almost 50% of all markers (6 out of 13—CD56, CD70, CD71, CD81, CD107a, and CD274) showing that application of CITE-seq extended antibody panels can reveal unusual surface markers that would otherwise be missed based on mere RNA profiling. Previously, CD56 expression was shown by immunohistochemistry staining in various tumors with neuroendocrine differentiation^74^, including melanoma^52,75,76^. CD56 is involved in cell–cell and cell–matrix interactions during development and differentiation and was implicated in homophilic and heterophilic interactions^77^. *NCAM1* gene expression did not correlate with CD56 protein abundance in the cell type-specific analysis. CD56 was furthermore predominantly found on “protein only” level in both liquid and solid cohorts and in an independent study^65^. To address the loss of spatial resolution and validate CD56 expression identified by CITE-seq, we performed multiplex immunohistochemistry coupled with advanced multispectral imaging. We profiled CD56 expression in a paired histological section of metastatic melanoma LN and identified clusters of MLANA-positive cells expressing CD56 that were organized in circular shapes. Previous studies have highlighted the possible role of CD56 as a predictive biomarker^78^, druggable target^79^, and its role in NK-cell mediated immunity^80^. In primary melanoma cultures, increased expression of CD56 was identified in cells showing statistically significant higher capability to cross the in vitro blood-brain-barrier model^81^ and all cultures established from brain metastases of melanoma showed CD56 expression^82^, suggesting its role in invasion and its potential importance as a biomarker in metastatic melanoma.

Limitations of CITE-seq analysis include the loss of surface epitopes due to enzymatic dissociation, capture of only pre-selected surface targets and loss of spatial resolution. These can be addressed by complementary spatial profiling, which is required to validate the findings and identify the biologically relevant spatial interactions. A limitation of our CITE-seq analysis using manual thresholds could be further optimized by using automated thresholding as recently proposed^55^, while general limitations of antibodies for protein detection in regard to restricted panels, binding specificity and cross-specificity should eventually be overcome by further developments in single-cell proteomics using mass spectrometry such as reported by SCoPE-MS^83^.

In this study, we demonstrated that CITE-seq can be applied to clinically relevant solid tissue biopsies and addressed common limitations that arise from enzymatic digestion. We demonstrated an optimization of the enzymatic digestion protocol for a complex solid tissue such as healthy skin and presented a simple method of applying thresholds on cell type-specific ridge plots to utilize large antibody panels for CITE-seq without prior titration steps. Finally, we applied the CITE-seq method for biomarker discovery, identified a potentially druggable surface protein CD56^84,85^ on metastatic melanoma cells, and validated its expression on a matched tissue. CITE-seq represents a powerful profiling modality that by virtue of its quantitative and qualitative dual transcriptome and surface proteome readouts allows global unbiased cell type composition analysis, RNA-protein correlations quantification as well as simultaneous DE and DA analysis, on RNA and protein levels, respectively.

## Methods

### Human primary tissue and live-cell biobanking

Human healthy skin, primary and melanoma metastasis samples, as well as PBMCs from consenting patients treated with immunotherapy were obtained from the Dermatology Biobank, University Hospital Zurich (BASEC Nr.2017-00494). Experimental and clinical information are summarized in Supplementary Data 2. After collection, tissue sample biopsies and surgical material were live-cell biobanked. Briefly, collected tissue samples were cut into small pieces of a maximum 2 × 2 mm and up to four pieces were placed into one cryovial (Sarstedt, cat. no. 72.380.002) containing 1 mL of freezing medium consisting of 90% FBS (Biowenst, cat. no. S006420E01, batch no. S169419181H), 10% DMSO (Sigma, cat. no. 102148154, filter-sterilized using 0.22 μM filter (Steriflip, Milipore, cat. no. SCGP00525)), slow-frozen in pre-chilled CoolCell® Containers (Corning, cat. no. 432001) and stored in −80 °C fridges within 30 min after collection.

PBMCs isolation: Buffy coats from healthy donors were obtained from Blutspende Zurich (Kunden No. 6561, Biobank project). PBMCs were isolated using a protocol described elsewhere (PBMC isolation and cryopreservation^86^) with minor modifications. Briefly, buffy coats diluted with PBS (Ca^2+^/Mg^2+^-free, Gibco, cat. no.10010-015) were layered onto HISTOPAQUE®−1077 (Sigma, 10771-500 mL) for healthy donor 1 and Ficoll-Paque-1084 (Cytiva, cat. no.17-5446-52) for healthy donors 2-3. Gradient centrifugation was performed at 760 g for 20 min with the brakes OFF. After collecting the mononuclear cell layer, cells were washed four times with PBS and spun down at 350 × *g* for 8 min at room temperature. Cell count and viability were accessed on Luna-FL^TM^ cell counter (Dual Fluorescence Cell counter, Logos Biosystems Inc., cat. no. L1001) using acridine orange propidium iodide (AOPI, Logos Biosystems Inc., cat. no. F23001) live/dead staining. Up to 10^7^ PBMCs were cryopreserved as described above in the live-cell biobanking section.

### Processing of cryopreserved solid tissue and enzymatic dissociation

Live-cell biobanked tissue samples were quickly thawed in a water bath set to 37 °C, re-suspended in 10 mL of ice-cold RPMI (Sigma, cat. no. R0883) with 0.04% BSA (Sigma-Aldrich, cat. no. A7906) and incubated for 10 min on ice to allow DMSO to diffuse from the tissue. Samples were spun down at 300 × *g* for 5 min and cut into small pieces with a scalpel. This step was performed to increase contact between the enzymes and the total surface area of the tissues in a small amount of enzyme mixture. Small tissue pieces were placed in the optimal enzymatic mixture and incubated for 30 min to 3 h at 37 °C with continuous rotation on a MACSmix tube rotator (Miltenyi Biotec, cat. no. 130-090-753) and trituration every 15 min using wide-bore pipet tips (Supplementary Note 1).

After incubation with the respective enzymatic mixture, digested tissue was sequentially filtered through 100 µm (Falcon, cat. no. 352360) and 35 µm cell strainers (Falcon, blue capped FACS tubes - cat. no. 352235). For samples with viability below 70% and when cell numbers allowed (>10^5^ cells total), apoptotic and dead cells were removed by immunomagnetic cell separation using the Annexin Dead Cell Removal Kit (StemCell Technologies, cat. no. 17899) and EasySep^TM^ Magnet (StemCell Technologies, cat. no. 18000). If the cell pellet appeared red, red blood cell (RBC) lysis was performed following the manufacturer’s instructions (Roche, cat. no. 11814389001). Briefly, the cell pellet was resuspended in 500 μL of RBC lysis buffer and incubated at room temperature for 3 min. Next, cells were washed with a resuspension buffer (PBS with 0.04% BSA), spun down and resuspended in a resuspension buffer. If the cell pellet appeared still red, RBC lysis was repeated. Cell number and viability were assessed on a Cellometer K2 Image Cytometer (Nexcelom Bioscience, cat. no. Cellometer K2) using ViaStain AOPI Staining Solution (Nexcelom Bioscience, cat. no. CS2-0106-5mL) and PD100 cell counting slides (Nexcelom Bioscience, cat. no. CHT4-PD100-003).

Optimization of healthy skin dissociation - experiment outline: we selected slow-frozen healthy skin biopsies of sufficient size that allowed for testing of three dissociation protocols on the same sample. In short, tissues were thawed and DMSO washed out. Biopsies were cut into three pieces and weighed on a precision scale. Each piece underwent a separate dissociation protocol as described in Supplementary Note 1 and Fig. 2a. Dead cell removal was performed whenever necessary and possible, and suspensions were stained with hashing antibodies whenever possible. Finally, samples were pooled and stained with a CITE-seq antibody cocktail.

Enzymatic digestion effect on PBMCs - experiment outline: three vials of PBMCs from healthy donors were thawed, counted, and split into three equal parts. Aliquot 1 was kept in PBS with 1% BSA and incubated for one hour at 37 °C (untreated), aliquot 2 was treated with SkinD for one hour at 37 °C while aliquot 3 was treated with TumorD protocol for one hour at 37 °C. Processing for antibody staining was performed as described below in the “Antibody staining” section. Donors 2 and 3 were processed on a separate day from Donor 1.

### Oligo-labeled antibody panels and staining

Oligo-labeled antibody panels were ordered from the BioLegend TotalSeq-C product line compatible with 10x Genomics 5P V(D)J immune profiling kits and concentrations were chosen according to manufacturer instructions for each antibody. The panel consisting of 97 antibodies (Supplementary Data 2) was pooled in a labeling buffer (PBS with 1% BSA). Antibody staining was performed similarly to the demonstrated 10x Genomics protocol Cell Surface Protein Labeling for Single Cell RNA Sequencing Protocols (https://assets.ctfassets.net/an68im79xiti/6p0emIeLO8bsxinEbKgcfF/275a5752f4e4347f75a1f649bd824463/CG000149_DemonstratedProtocol_CellSurfaceProteinLabeling_RevB.pdf), omitting dextran sulfate staining as recommended by BioLegend (https://www.biolegend.com/en-us/protocols/totalseq-b-or-c-with-10x-feature-barcoding-technology). After Fc receptor blocking, samples were first stained with hashing antibodies (BioLegend, see Supplementary Data 2) in ~100 µL volume for 30 min on ice. Afterwards, cells were washed three times with labeling buffer (PBS with 1% BSA) before incubation in a final pool of oligo-barcoded BioLegend TotalSeq-C antibodies in 100 µL volume for another 30 min. Finally, cells were washed three times and resuspended in a resuspension buffer (PBS with 0.04% BSA). Cell number and viability were profiled as described in the above section (Processing of cryopreserved tissue and enzymatic digestion) and optimal cell concentrations were set according to 10x Genomics protocols (700-1200 cells/µL).

### Single-cell droplet generation and processing

Stained cell suspensions were loaded and processed using the 10x Genomics Chromium platform with the 5P V(D)J immune profiling kit on 10x Genomics Chromium Single Cell Controller (10x Genomics, PN-120263). Hashed samples were super-loaded with 9,000 to 20,000 cells per lane (see Supplementary Data 2). GEX and SPEX libraries were amplified and sequenced on the Illumina NextSeq 500 or NovaSeq 6000 platform at recommended sequencing depth (20,000-50,000 reads/cell for GEX libraries, and >7000 reads/cell for SPEX libraries).

### Multiplex immunohistochemistry staining and multispectral imaging (Akoya Vector Polaris)

Histological slides were stained with primary (at 1:100 concentration) and opal antibody pairs on a Leica Bond RXm following manufacturer instructions supplied from Akoya (Table 1). Prior to scanning, the bottom of the slides was cleaned with 70% ethanol. Multispectral slide scanning was performed on the Vectra Polaris at ×40 magnification. Regions of interest (ROIs) were annotated with PhenoChart and spectral unmixing and cell segmentation were performed with inForm 2.4.9. Raw QPTIFF images were exported as TIFF files.

**Table 1:** Antibodies and Opal reagents for mIHC.

Primary	Opal
MelanA (NBP1-30151, NovusBio)	Opal 780 (NEL871001KT)
CD68 (ab213363, Abcam)	Opal 690 (NEL871001KT)
CD56 (ab220360, Abcam)	Opal 520 (NEL871001KT)
CD8 (ab4055, Abcam)	Opal 620 (NEL871001KT)

### PBMCs stimulation and flow cytometry

Live-frozen PBMCs from three patients receiving immunotherapy (Supplementary Data 2) were thawed by drop-wise resuspension in media (RPMI 1640 (Sigma-Aldrich, cat. no. R0883) supplemented with 5 nM L-glutamine (Gibco, Thermo Scientific, cat. no. 25030-024), 1 mM sodium pyruvate (Sigma-Aldrich, cat. no. S8636), 10% heat-inactivated fetal bovine serum (Biowest, cat. no. S181H) and 1% Pen-Strep (Gibco, Thermo Scientific, cat. no. 15140-122)) and rested on ice for 10 min. Cells were counted with AOPI (Logos Biosystems, cat. no. F23002) and viability was found to be over 90%. Cells were seeded in round bottom 96 well plates in media at 0.5 Mio cells/well and incubated with or without cell stimulation/protein export inhibitor cocktail (eBioscience, cat. no. 00-4975-93) for 4 h at 37 °C and 5% CO_2_. Next, cells were washed with PBS with 2% FCS, and stained with anti-human CD3 (APC, BioLegend, cat. no. 317318, stock concentration 50 µg/ml, dilution 1:50, final concentration 1 µg/ml), anti-human CD4 (PerCP, BioLegend, cat. no. 317432, stock concentration 200 µg/ml, dilution 1:50, final concentration 5 µg/ml), anti-human CD8 (PE-Cy7, BioLegend, cat. no. 344712, stock concentration 100 µg/ml, dilution 1:100, final concentration 1 µg/ml), anti-human CTLA-4 (Biolegend, clone BNI3, APC/Fire 750, cat. no. 369627, stock concentration 25 µg/ml, dilution 1:20, final concentration 1.25 µg/ml) or Mouse IgG2a, κ Isotype control (APC/Fire 750, Biolegend, cat. no. 400283), anti-human CTLA-4 (PE, Biolegend, clone L3D10, cat. no. 349905, stock concentration 200 µg/ml, dilution 1:20, final concentration 10 µg/ml) or PE Mouse IgG1, κ Isotype control (PE, Biolegend, cat. no. 400113). Samples were analyzed on a LSRFortessa™ Cell Analyzer (BD Biosciences). Isotype controls were used at the same concentrations as the matched antibodies. Compensation was performed with UltraComp eBeads™ (Invitrogen, cat. no. 01-2222-42). The flow cytometry data were analyzed in FlowJo™ v10.0.8.

### Data analysis

All CITE-seq data analysis was embedded in a workflow for single-cell GEX and SPEX analysis as well as hashing analysis^35^. In the following, we describe in brief the main steps of the sequencing data analysis.

Preprocessing and hashing analysis: Raw reads were mapped to the GRCh38 reference genome using 10x Genomics Cell Ranger 3.1.0 to infer read counts per gene per cell. GEX and SPEX libraries were processed independently, and the Cell Ranger “–force-cells” option was set to initially retrieve the number of loaded cells per experiment. For samples sequenced on the NovaSeq platform, index-hopping removal using a method developed by Griffiths et al. was performed^87^. Next, hashed samples were demultiplexed by first applying CITE-seq count in order to count hashtags per cell and subsequent normalization and hashtag assignment using Seurat^88^.

GEX analysis: After hashtag-based demultiplexing of samples, GEX data of each sample was analyzed using the scAmpi workflow^89^. In brief, UMI counts were quality controlled and cells and genes filtered to remove known contaminants: cells where over 50% of the reads mapped to mitochondrial genes and cells with fewer than 400 different expressed genes were removed, as well as non protein-coding genes and genes that were expressed in <20 cells. The 400 DEG was chosen as the default threshold and is in place to remove cells with low gene diversity, as those “cells” have an increased risk of being artifacts. Subsequently, counts were normalized and corrected for cell-cycle effects and library size using sctransform^90^. Similar cells were grouped based on unsupervised clustering using Phenograph^91^ and an automated cell type classification was performed independently for each cell^92^. Cell type annotation was performed using lists of cell type defining highly expressed genes from previous publications as follows (Supplementary Data 2): for lymph node melanoma metastasis and healthy skin samples, gene sets from Tirosh et al.^93^ and Tabib et al.^94^ were used, respectively. For primary cutaneous melanoma samples, gene sets from Tirosh et al.^93^ were enriched with keratinocyte gene sets from Tabib et al.^94^. Healthy PBMC data was annotated using gene lists from Zhang et al.^95^ and Newman et al.^96^ and enriched with dendritic cell gene sets from Villani et al.^97^. The label “unknown” was assigned if for a particular cell none of the compared cell type marker lists showed sufficient similarity to the gene expression profile of the cell. In contrast, a cell was labeled “uncertain”, if the expression profile of the cell is sufficiently similar to more than one marker list (i.e., the *p*-values returned by the Mann–Whitney *U*-Test performed by the scROSHI cell typing method^92^ are close to the most similar cell type and the second most similar cell type).

SPEX processing: preprocessing, thresholding and visualization: Initial SPEX counts per cell were determined using 10x Genomics Cell Ranger 3.1.0 (Hohhm/CITE-seq-Count: 1.4.2 - https://zenodo.org/record/2590196#.YWcNFmQza2x). Raw counts were log-transformed and visualized in a cell type-specific expression ridge plot to allow manual threshold definition. Similar to, e.g., FACS experiments, the observed raw counts contain background noise, which can be removed using manually selected thresholds (for a manually set threshold overview, refer to Supplementary Data 1). Based on the thresholds, only cells with a SPEX count exceeding the threshold were determined as positive for the respective antibody (Supplementary Data 2). GEX and SPEX counts were combined to calculate a UMAP embedding that displays both GEX and SPEX-based effects. In addition, we performed a clustering based on combined GEX and SPEX counts using BREM-SC^98^. To inform on cell type-dependent SPEX counts, ridge plot visualization was performed on a per cell type level.

Cohort integration: Samples originally part of the same hashing experiment are assumed to show no sequencing or antibody staining batch effects, as they have been pooled together before processing. Thus, when grouping samples from the same hashing experiment, no additional batch correction was performed. Instead, after grouping the individual samples, the original GEX and SPEX counts from the single samples are visualized on the combined UMAP.

When integrating samples across hashing experiments the top 3000 genes observed as variables in most samples were used as anchors; additional batch correction was applied using the Seurat CCA method^99^ and multimodal integration was performed using the Seurat WNN method^88^.

Differential gene expression and protein abundance analysis: To compare the effect of different treatment protocols on PBMC samples (untreated versus SkinD versus TumorD) a differential gene expression and surface protein abundance analysis was performed on the integrated samples. To avoid any bias due to sample composition differences across the compared groups, the DE analysis was performed by major cell types as follows: Per cell type subset, expression levels were compared using Seurat FindMarkers with a significance level of FDR < 0.0001 and |logFC| > 3 for GEX data and FDR < 0.0001 and |logFC| > 0.5 for SPEX. Specifically for SPEX data, the differential expression analysis is performed on un-thresholded counts, as existing tools are not built to be aware of thresholding; the final results are visualized however throughout the manuscript on the thresholded counts (Supplementary Data 1) to reduce the noise and highlight expression differences (Supplementary Fig. 1c and d), see also Grob et al.^35^.

GEX and SPEX codetection analysis: To compare the detection of GEX and SPEX counts, the average of the fraction of cells was calculated when (i) positive for both RNA and associated surface protein, (ii) negative for both RNA and surface protein, (iii) positive for RNA but negative for surface protein, and (iv) negative for RNA but positive for surface protein. A cell was identified as “positive” for a RNA if the UMI count was greater than zero. A cell was identified as “positive” for a surface protein if the UMI count was greater than the predefined threshold (see Supplementary Data 1). The comparison was performed across six liquid biopsy samples (three healthy and three immunotherapy-treated PBMCs) and across all solid biopsy samples (five healthy skin samples, three primary melanomas and three metastatic melanomas from the lymph node). For the three categories (“RNA only”, “protein only” and “RNA and protein”), feature counts were converted into percentages. Features belonged predominantly to one category, if >50% of cells were assigned to one category. In case the threshold was not reached for a single category, the feature was labeled as “other” (Supplementary Data 1).

RNA-protein correlation analysis: We calculated RNA-protein correlation coefficients on aggregated sample and cell levels: *Aggregated sample level:* In brief, counts were aggregated by samples and a matrix of counts for all RNAs and proteins (antibody-derived oligos) was created for each sample. RNAs or proteins without counts were excluded from the analysis. Next, RNA-protein pairs were filtered, keeping only the relevant ones as shown in the RNA-protein match lookup table (Supplementary Data 2). The Pearson correlation was computed on the matrices, obtaining both the correlation coefficient and the significance level for every possible pairing. *Cell level:* the expression of RNA and protein abundance was extracted from each cell, the Pearson correlation was computed using each cell as a separate observation and followed by multiple testing correction using the Bonferroni method on the *p*-values.

The results were visualized in the form of barplots for all detected RNA-protein pairs of the sorted correlation coefficients, with color coding for the p-values: gray-to-blue gradient representing *p*-values below threshold; blue-to-red gradient representing *p*-values above thresholds; the red dotted lines show the threshold (*p* < 0.05) for both the positive and anti-correlated pairs. Pairs were positively correlated if the expression of both RNA and protein in the available observations showed a tendency to change in the same direction, i.e. a higher observed expression of one feature in the pair corresponded to a higher observed expression of the other feature. Anti-correlation was assigned to RNA-protein pairs if the change in expression of one feature was opposite to the changes observed in the other feature, i.e., a higher observed expression in either the RNA or the protein corresponding to a lower observed expression of the other feature.

Single-cell spatial image analysis: Spatial expression analysis using Giotto (version 2.0.0.957) was performed as follows. Cell segmentation data output from inForm image processing was imported via the createGiottoObject function. The giotto object was further filtered using filterGiottowith the following argument thresholds:expression_threshold = 1, feat_det_in_min_cells = 3, min_det_feats_per_cell = 1. Normalization was applied using normalizeGiotto with the default scale factor of 6000, log_norm = FALSE, library_size_norm = FALSE, scale_feats = FALSE, and scale_cells = TRUE. Dimensionality reduction was performed with runPCA then runUMAP. Clustering was performed with doLeidenCluster with a resolution of 0.2 and 100 iterations. Clusters with similar expression patterns were merged and a final number of 5 clusters was chosen. Clusters were named CD56+/MLANA+, CD68, CD8, MLANA, and Unknown based on the average expression of the markers. Cell identities were mapped back to the original image using ggplot2.

### Statistics and reproducibility

In the following, we provide a summary of the statistical analysis performed throughout this manuscript. Further details are available as part of the methods.

GEX analysis included counts normalization, library size, and cell-cycle effect correction using sctransform^90^. Cell type assignment was based on the p-values returned by the Mann–Whitney *U*-Test performed by the scROSHI cell typing method^92^. Combined GEX and SPEX counts clustering was performed using BREM-SC^98^. Cohort integration included additional batch correction using the Seurat CCA method^99^ and multimodal integration using the Seurat WNN method^88^. Differential expression analysis was performed using Seurat FindMarkers with a significance level of FDR < 0.0001 and |logFC| > 3 for GEX data and FDR < 0.0001 and |logFC| > 0.5 for SPEX. Specifically for SPEX data, the differential expression analysis is performed on un-thresholded counts, as existing tools are not built to be aware of thresholding. RNA-protein correlation was performed by calculating the Pearson correlation on the count matrices, obtaining both the correlation coefficient and the significance level for every possible pairing. The significance threshold was set to *p* < 0.05.

Patients’ samples were used for healthy skin, primary melanoma, metastatic melanoma, healthy and immunotherapy-treated PBMCs to reach statistical power in our investigations. The explorative nature of the study did not permit estimating effect sizes prior to the analysis. Healthy skin samples were pre-processed, divided into equal parts, and randomly assigned to one of the digestion protocols. All the CITE-seq samples passed the QC of the 10x Cell Ranger data analysis pipeline. In flow cytometry experiment, *n* = 3 represents biologically independent PBMCs samples.

### Reporting summary

Further information on research design is available in the Nature Portfolio Reporting Summary linked to this article.

### Supplementary information

Supplementary information

Description of Additional Supplementary Files

Supplementary Data 1

Supplementary Data 2

Reporting Summary

## Data Availability

The scRNA-seq data generated in this study has been deposited to the European Genome-phenome Archive (EGA) database under accession code EGAS00001005849. Source data is available from Supplementary Data 1.

## Code availability

The scAmpi workflow^89^ for GEX analysis is available on github: https://github.com/ETH-NEXUS/scAmpi_single_cell_RNA, as well as the gExcite workflow^35^ for GEX, SPEX, and hashing analysis: https://github.com/ETH-NEXUS/gExcite_pipeline. The code used for the downstream analysis is available on GitLab: https://gitlab.ethz.ch/nexuscbu/cite-seq_method_paper. The custom scripts used for the paper are on Zenodo^100^.
