# Supplementary material for: Dynamic thresholding and tissue dissociation optimization for CITE-seq identifies differential surface protein abundance in metastatic melanoma
Source: Commun Biol. 2023 Aug 10;6:830. doi: 10.1038/s42003-023-05182-6 (PMC10415364; doi:10.1038/s42003-023-05182-6)
Supplement: Supplementary file 1 — Supplementary information [file 42003_2023_5182_MOESM1_ESM.pdf]

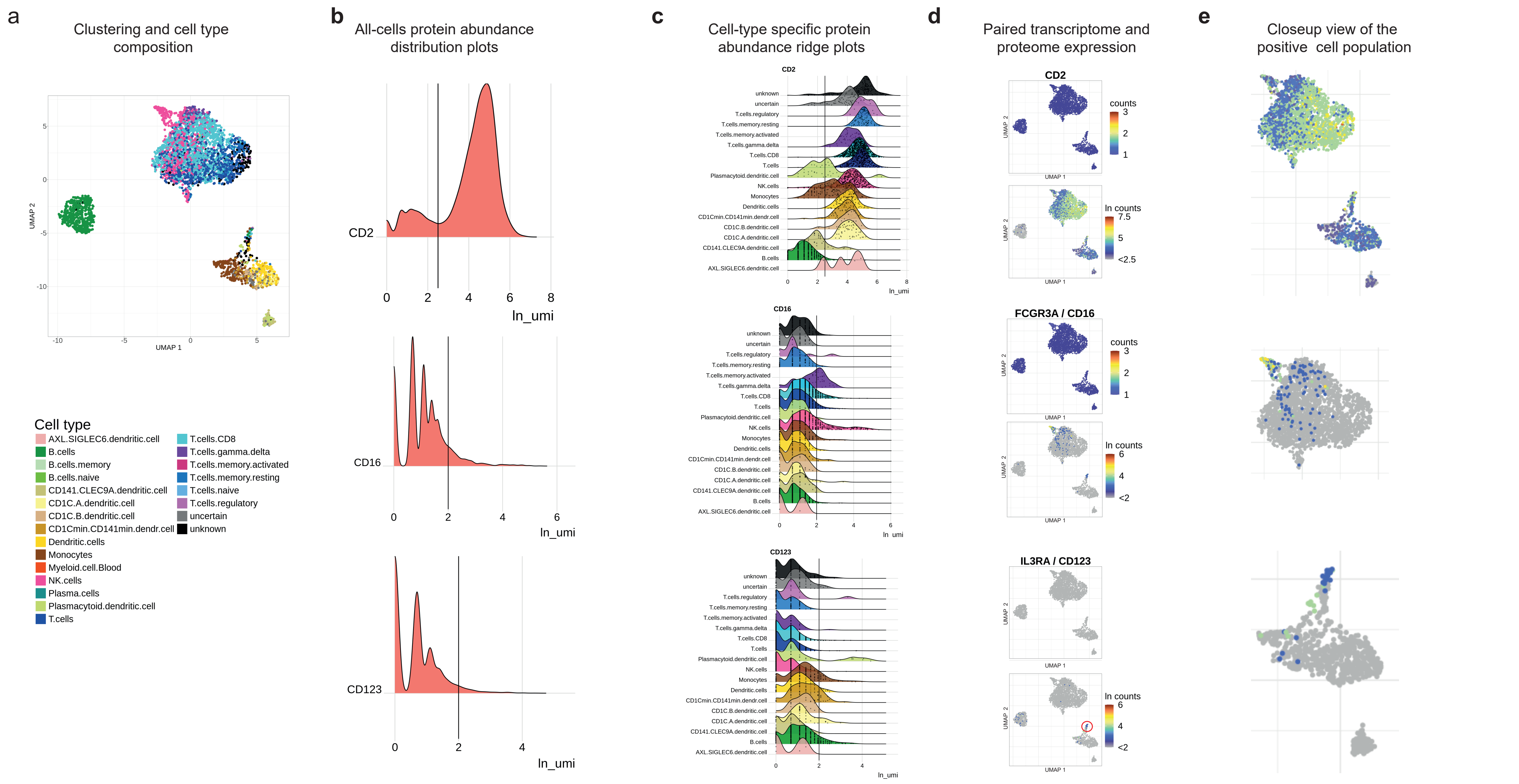

Supplementary Figure 1: Cell type-specific protein abundance ridge plots for marker detection on rare cell types and manual thresholding.
a Exemplary analysis of a healthy PBMC patient (Donor 1) showing UMAP clustering and annotation of cell types. In comparison to **b** all cells protein abundance distribution plots **c** cell type-specific protein abundance ridge plots detect widely expressed markers such as CD2 on lymphocytes and signals from rare cell types such as CD16 on NK cells or CD123 on plasmacytoid dendritic cells. Manually set thresholds are shown as vertical bars. **d** Paired transcriptome and proteome expression UMAP plots for selected markers and **e** a close-up view of the positive cell type in respective protein expression UMAP.

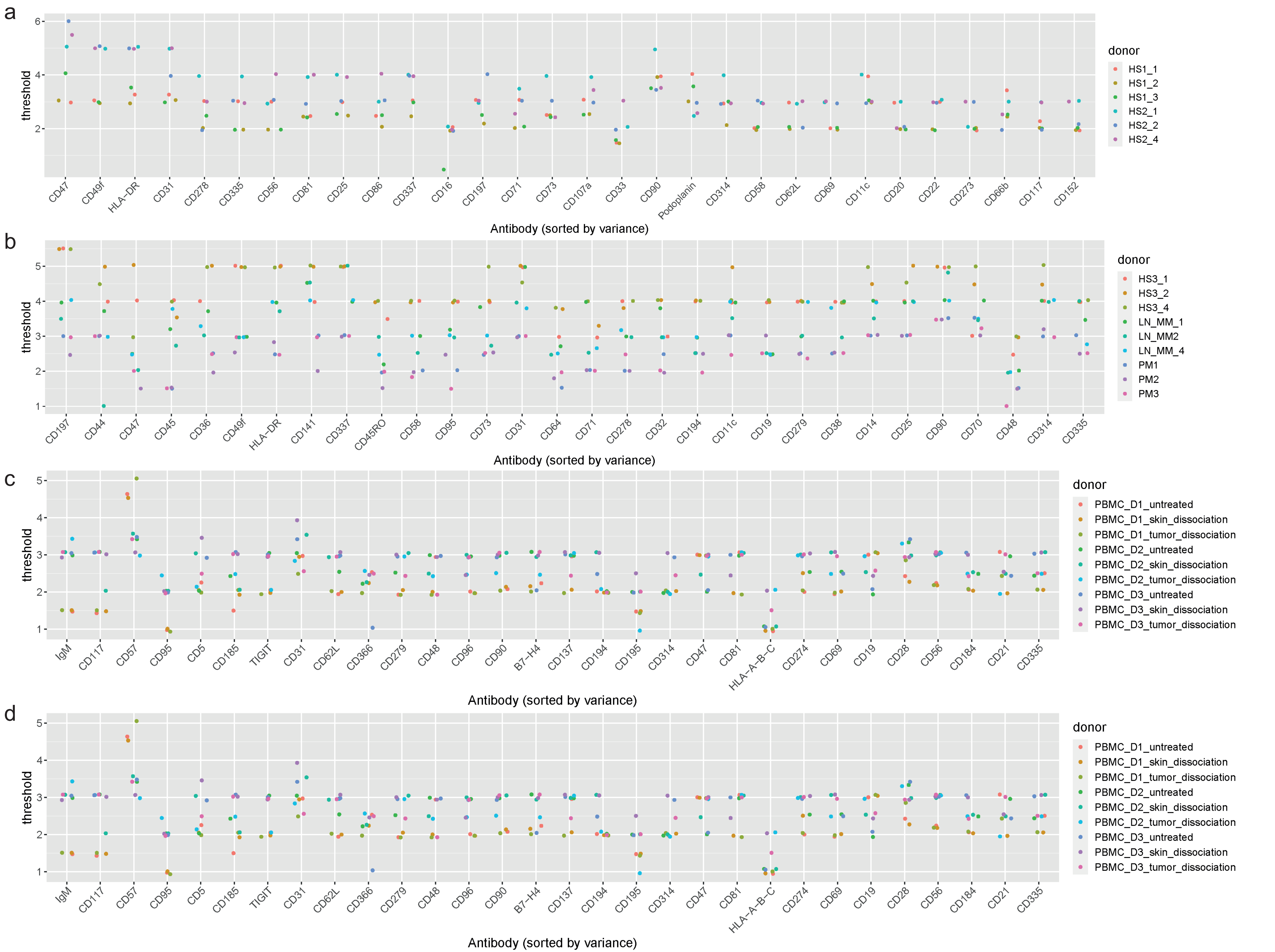

Supplementary Figure 2: **Antibody threshold distribution.** Scatterplots showing the manual thresholding values assigned to the 30 antibodies with largest threshold value variance across selected samples with antibody signals sorted from largest to smallest variance. **a** Healthy skin optimization (6 samples, 2 donors). **b** Solid tissue cohort (9 samples, 9 donors). **c** Liquid cohort for healthy and immunotherapy-treated PBMCs (6 samples, 6 donors). **d** PBMC model for untreated PBMCs, SkinD- and TumorD-enzymatically treated PBMCs (9 samples, 3 donors).

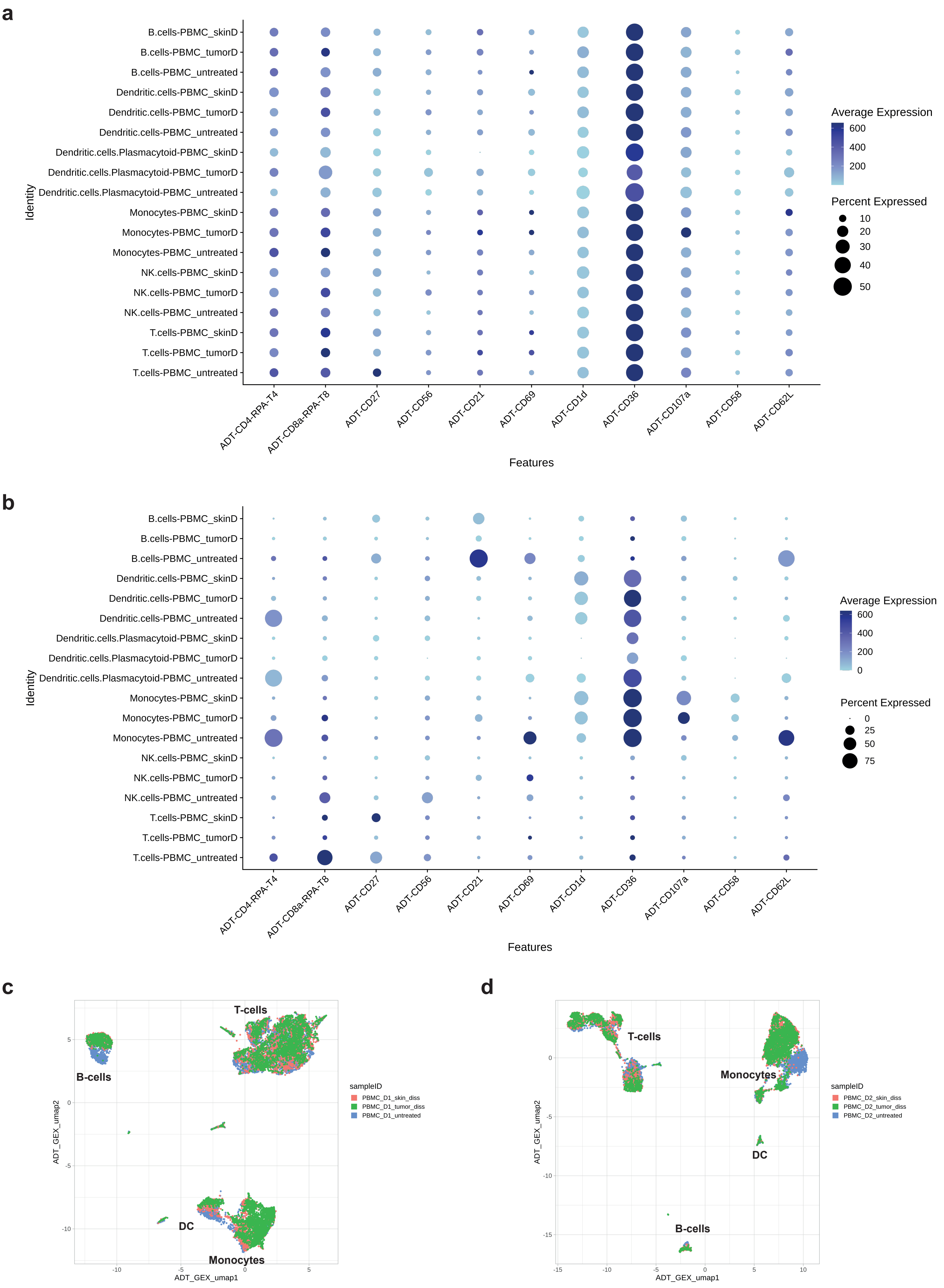

Supplementary Figure 3: Digestion protocol specific protein abundance and cell clustering. Selected marker protein abundance dot plots before **a** and after **b** application of thresholds applied to PBMC model cell types and samples. UMAP plots from Donor 1 **c** and Donor 2 **d** showing protocol-specific influence on clustering of different cell types.

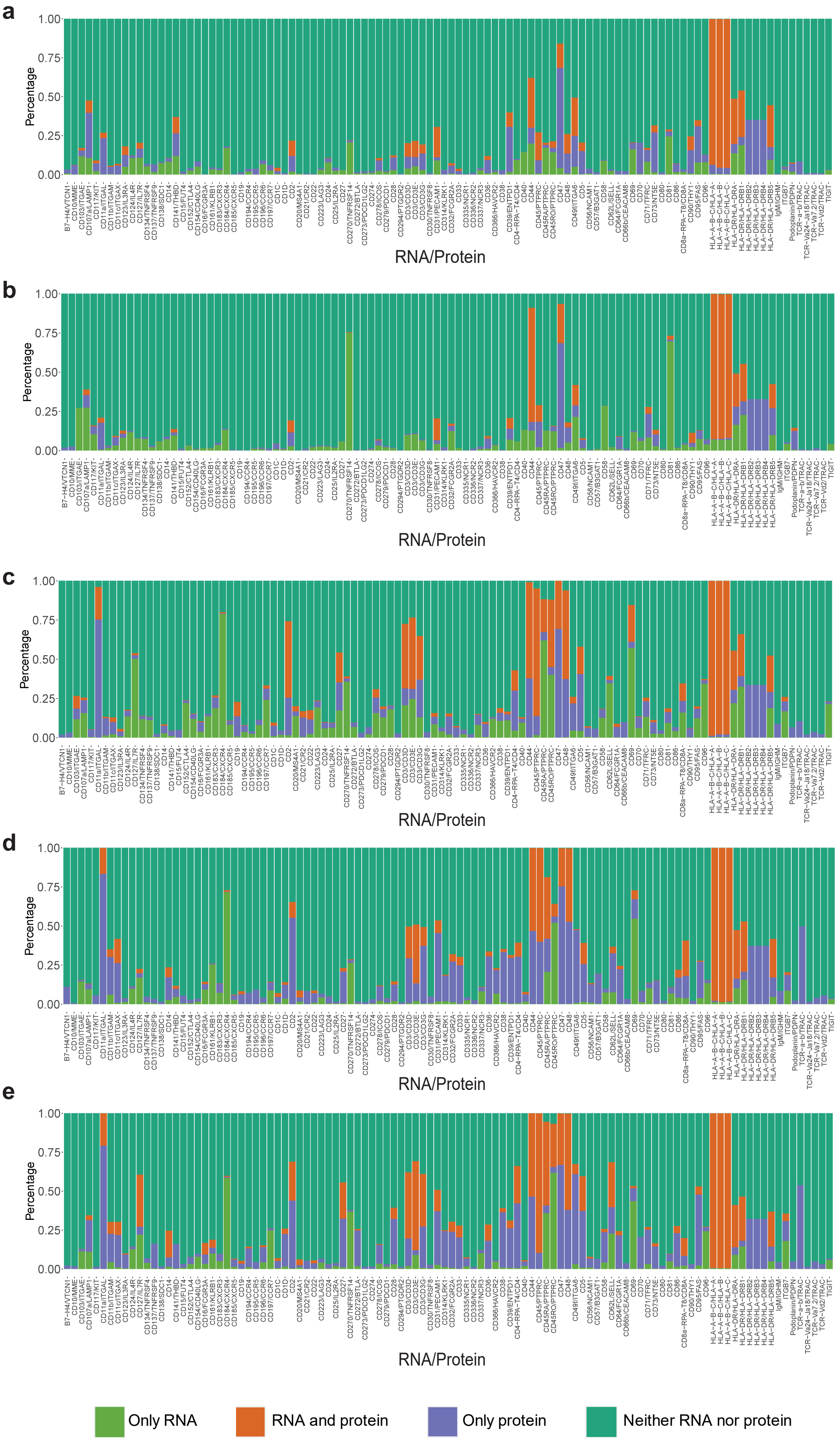

Supplementary Figure 4: RNA and protein codetection plots. Plots showing percentage of cells with “RNA only”, “RNA and protein”, “protein only” or “neither RNA nor protein” detection. Feature codetection plots of **a** healthy skin (n=5, biologically independent samples), **b** primary melanoma (n=3, biologically independent samples), **c** lymph node metastatic melanoma samples (n=3, biologically independent samples), **d** PBMCs from healthy donors (n=3, biologically independent samples, no digestion) and **e** PBMCs from immunotherapy-treated melanoma patients (n=3, biologically independent samples).

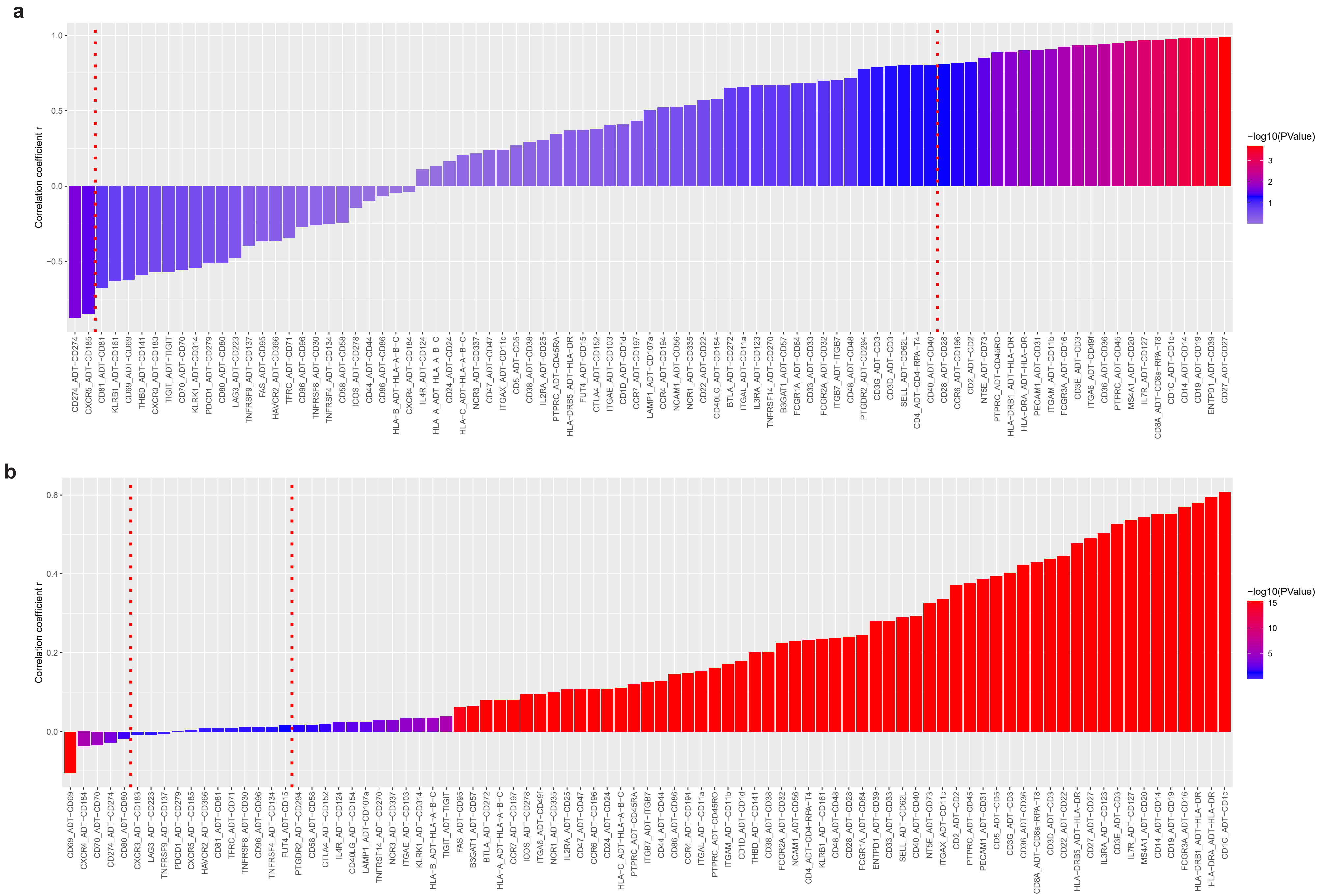

Supplementary Figure 5: RNA and protein expression correlation. Pearson correlation coefficients of 87 RNA-protein pairs on aggregated **a** sample level (n=6, biologically independent samples) and **b** cell level (n=16,142 single cells) for the liquid biopsy cohort. The Pearson correlation coefficient between each RNA-protein pair is shown along with its related p-value. The significance threshold is set to 0.05 and indicated as a red, dotted vertical line. Pairs were excluded if either one or both members of the pair were not detected (Supplementary Data 5 and 6).

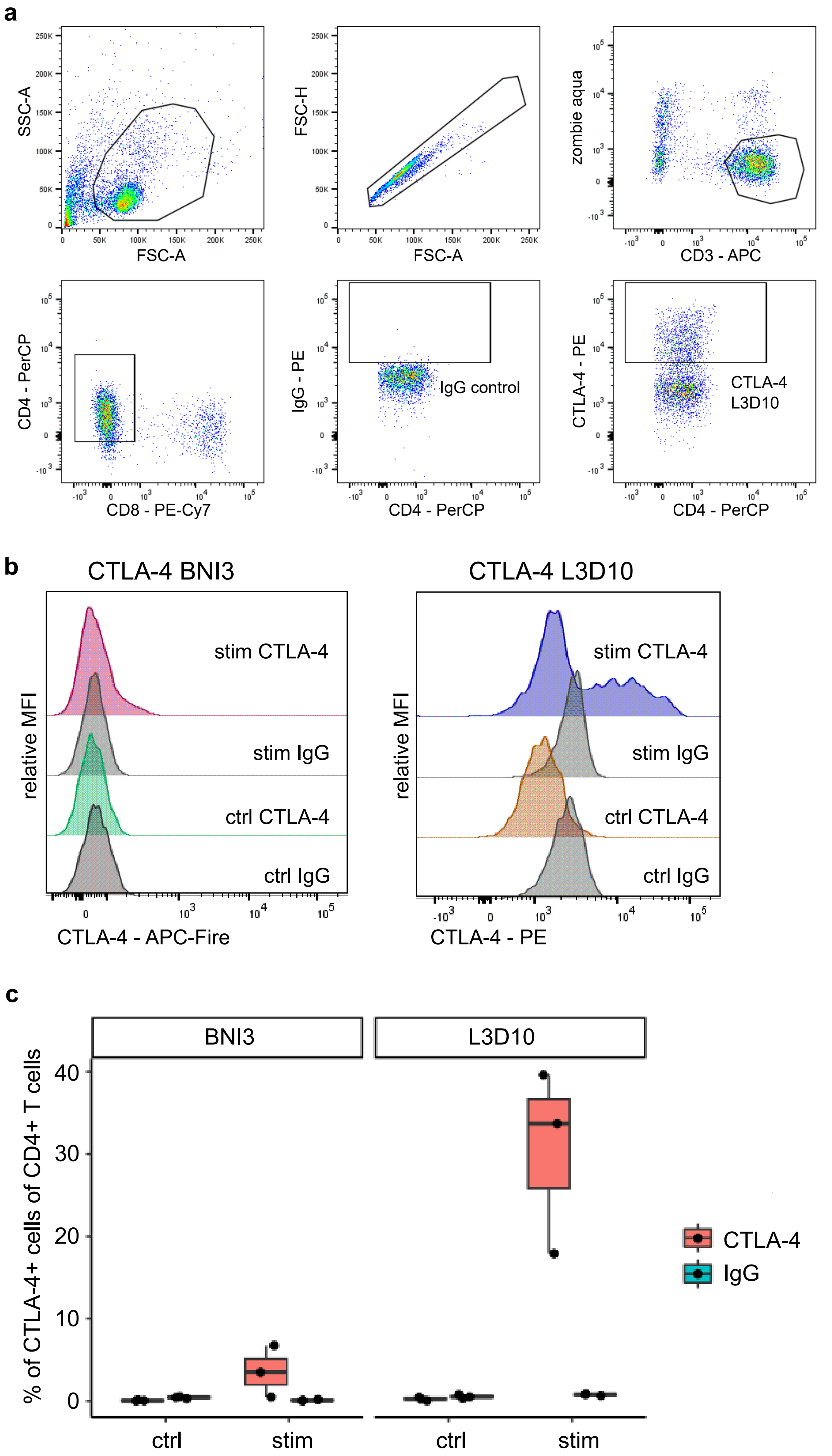

Supplementary Figure 6: CTLA-4 expression on peripheral blood CD4+ T cells.
a Cells were gated as lymphocytes/single cells/CD3+/CD4+CD8- and gates for CTLA-4 were adjusted according to the matching isotype control. **b** relative mean fluorescence intensity (MFI) from two different anti-CTLA-4 antibody clones (BNI3 or L3D10) or the respective isotype controls on CD4+ T cells, either unstimulated cells (ctrl) or PMA/ionomycin stimulated cells (stim). **c** percentage of CTLA-4+ of CD4+ T cels in the indicated conditions, boxplots represent median and 25th and 75th percentile, whiskers extend to the lowest and largest values, n=3 biologically independent samples.

Supplementary Note 1: Digestion protocols by tissue type tested with CITE-seq.

Tissue type	Protocol	Protocol description	
Healthy skin	Protocol D/C/T (main protocol: SkinD) - sequential digestion protocol (3 steps)	Steps	Incubation time
		Step 1: Dissociate tissue pieces in 5 mL of 2.4 U Dispase II (Roche, cat. no. 04942078001) dissolved in a resuspension buffer consisting of PBS with 0.04% BSA.<br><br>Add tissue pieces and triturate with a wide-bore pipette every 15 minutes.	up to 2 hours at 37°C
		Step 2: Spin down pre-digested tissue and resuspend the pellet in a mixture containing 1000 U Collagenase IV (Worthington, LS004188), 15 KU DNAse I (Sigma, cat. no. D5025) and 2 mM $CaCl_2$ (Sigma, cat. no. 746495). Top up to 5 mL with a resuspension buffer.<br><br>Triturate with a wide-bore pipette every 15 minutes.	30-60 minutes at 37°C
		Step 3: Pass pre-digested tissue suspension through a 100 µm cell strainer and keep aside on ice. Place the cell strainer with remaining undigested tissue pieces in a petri dish and add ~3 mL of 0.25% Trypsin-EDTA (Gibco, cat. no. 25200-56).<br><br>NB! Wash out EDTA at least 3 times before continuing to process for single-cell droplet generation.	10 minutes at 37°C
Healthy skin	Protocol D/CP - sequential digestion protocol (3 steps) modified from D/C/T protocol	Step1: Same as Step 1 in D/C/T protocol	up to 2 hours at 37°C
		Step 2: Spin down tissue and resuspend the pellet in enzymatic mixture:<br>Protease from *Bacillus licheniformis* (Sigma, cat. no. P5380 at 10 U/mL), 15 KU DNAse (Sigma, cat. no. D5025) and 2 mM $CaCl_2$ (Sigma, cat.no. 746495), top up to 5 mL with resuspension buffer.<br><br>Triturate with a wide-bore pipette every 15 minutes.	45 minutes on ice

		Step 3: Same as in protocol D/C/T.	10 minutes at 37°C
Healthy skin	Protocols MACS and MACS^{M} - Miltenyi MACS Human Whole Skin Dissociation Kit (Miltenyi Biotec, cat. no. 130-101-540)	Step 1:<br>We followed the manufacturer's instructions with (MACS^{M}) or without (MACS) steps involving gentleMACS™ Octo Dissociator (Miltenyi Biotec, cat. no. 130-095-937) (see step 2.2.8-10 in the protocol[1]). As recommended by Miltenyi, the use of Enzyme P was omitted to preserve surface cell epitopes (see step 2.2.3[1]).	According to manufacturer's instructions
Healthy skin	Protocol Liberases^{DH}	Step 1:<br>Liberases DH (0.28 Wünsch units/mL, Roche, cat. no. 5401054001), 15 KU DNAse (Sigma, cat. no. D5025) and 2 mM $CaCl_2$ (Sigma, cat.no. 746495), top up to 5 mL with resuspension buffer.<br>Triturate with a wide-bore pipette every 15 minutes.	Up to 2 hours at 37°C<br>Fill up 5mL Eppendorf tube with the enzymatic mixture
		Step 2: Same as Step 3 in protocol D/C/T.	10 minutes at 37°C
Primary and metastatic melanoma	Protocol TumorD - sequential digestion protocol (2 steps) established in Restivo *et al.*[2].	Step 1:<br>Enzymatic digestion mix:<br>5000 U Collagenase IV (Worthington, cat. no. LS004188), 15 KU DNAse I (Sigma, cat. no. D5025), 2 mL Accutase (Sigma, cat. no. A6964), dissolved in a total volume of 5 mL MACS tissue storage solution (Miltenyi Biotec, cat. no. 130-100-008) with 2 mM $CaCl_2$ (Sigma, cat.no. 746495).<br>Triturate with a wide-bore pipette every 15 minutes.	30-60 minutes at 37°C
		Step 2: Same as in Step 3 in protocol D/C/T.	10 minutes at 37°C

Supplementary References

1. https://www.miltenyibiotec.com/upload/assets/IM0007590.PDF
2. Restivo, G. *et al.* Live slow-frozen human tumor tissues viable for 2D, 3D, ex vivo cultures and single-cell RNAseq. *Commun. Biol.* **5**, 1144 (2022).
