# Supplementary material for: Dynamic thresholding and tissue dissociation optimization for CITE-seq identifies differential surface protein abundance in metastatic melanoma
Source: Commun Biol. 2023 Aug 10;6:830. doi: 10.1038/s42003-023-05182-6 (PMC10415364; doi:10.1038/s42003-023-05182-6)
Supplement: Supplementary file 2 — Description of Additional Supplementary Files [file 42003_2023_5182_MOESM2_ESM.pdf]

Description of Additional Supplementary Files

File name: Supplementary Data 1
Description: The source data used for th graphs and plots: Fig 2b, 2d, 3a, 3d-e, 4c, 5-6, 7b, 8a-b, 8e

File name: Supplementary Data 2
Description: Experimental and clinical summary of study samples. CITE-seq antibody panel used in this study. Detected antibodies per sample and experiment. RNA-protein match lookup. Marker s used for cell typing.
