# Supplementary material for: Dynamic thresholding and tissue dissociation optimization for CITE-seq identifies differential surface protein abundance in metastatic melanoma
Source: Commun Biol. 2023 Aug 10;6:830. doi: 10.1038/s42003-023-05182-6 (PMC10415364; doi:10.1038/s42003-023-05182-6)
Supplement: Supplementary file 5 — Reporting Summary [file 42003_2023_5182_MOESM5_ESM.pdf]

nature portfolio

Corresponding author(s): Aizhan Tastanova
Mitchell P. Levesque

Last updated by author(s): Jul 25, 2023

Reporting Summary

Nature Portfolio wishes to improve the reproducibility of the work that we publish. This form provides structure for consistency and transparency in reporting. For further information on Nature Portfolio policies, see our Editorial Policies and the Editorial Policy Checklist.

Statistics

For all statistical analyses, confirm that the following items are present in the figure legend, table legend, main text, or Methods section.

n/a	Confirmed	
☐	☒	The exact sample size (*n*) for each experimental group/condition, given as a discrete number and unit of measurement
☐	☒	A statement on whether measurements were taken from distinct samples or whether the same sample was measured repeatedly
☒	☐	The statistical test(s) used AND whether they are one- or two-sided<br>*Only common tests should be described solely by name; describe more complex techniques in the Methods section.*
☐	☒	A description of all covariates tested
☐	☒	A description of any assumptions or corrections, such as tests of normality and adjustment for multiple comparisons
☒	☐	A full description of the statistical parameters including central tendency (e.g. means) or other basic estimates (e.g. regression coefficient) AND variation (e.g. standard deviation) or associated estimates of uncertainty (e.g. confidence intervals)
☒	☐	For null hypothesis testing, the test statistic (e.g. *F*, *t*, *r*) with confidence intervals, effect sizes, degrees of freedom and *P* value noted<br>*Give P values as exact values whenever suitable.*
☒	☐	For Bayesian analysis, information on the choice of priors and Markov chain Monte Carlo settings
☒	☐	For hierarchical and complex designs, identification of the appropriate level for tests and full reporting of outcomes
☒	☐	Estimates of effect sizes (e.g. Cohen's *d*, Pearson's *r*), indicating how they were calculated

Our web collection on statistics for biologists contains articles on many of the points above.

Software and code

Policy information about availability of computer code

Data collection	CellRanger 3.1 developed by 10xGenomics was used to analyze raw scRNA sequencing files. Spectral unmixing and cell segmentation were performed with inForm 2.4.9. Raw QPTIFF images were exported as TIFF files for mIHC.
Data analysis	All software used in this manuscript have been described in the Methods section "Data Analysis". The scAmpi workflow for GEX analysis is available on github: https://github.com/ETH-NEXUS/scAmpi_single_cell_RNA, as well as the gExcite workflow for GEX, SPEX, and hashing analysis: https://github.com/ETH-NEXUS/gExcite_pipeline. The code used for the downstream analysis is available on GitLab: https://gitlab.ethz.ch/nexuscbu/cite-seq_method_paper. The custom scripts used for the paper is on Zenodo under the DOI: https://doi.org/10.5281/zenodo.8124136.

For manuscripts utilizing custom algorithms or software that are central to the research but not yet described in published literature, software must be made available to editors and reviewers. We strongly encourage code deposition in a community repository (e.g. GitHub). See the Nature Portfolio guidelines for submitting code & software for further information.

Data

Policy information about availability of data

All manuscripts must include a data availability statement. This statement should provide the following information, where applicable:

- Accession codes, unique identifiers, or web links for publicly available datasets
- A description of any restrictions on data availability
- For clinical datasets or third party data, please ensure that the statement adheres to our policy

The scRNA-seq data generated in this study has been deposited to the European Genome-phenome Archive (EGA) database under accession code EGAS00001005849. Source data is available from Supplementary Data 1.

Research involving human participants, their data, or biological material

Policy information about studies with human participants or human data. See also policy information about sex, gender (identity/presentation), and sexual orientation and race, ethnicity and racism.

Reporting on sex and gender	The sex of corresponding patients' samples is indicated in Supplementary Data 1 when available.
Reporting on race, ethnicity, or other socially relevant groupings	No race, ethnicity, or other socially relevant groupings are reported in the manuscript.
Population characteristics	This study used surplus and archival samples collected and stored in Dermatology Biobank of the University Hospital of Zurich. Only material from consenting patients was used for research purpose.
Recruitment	Only surplus material from consenting patients were included.
Ethics oversight	Cantonal ethical approval: Dermatology Biobank of Univeristy Hospital of Zurich: EK-687 and 800, KEK 2017-00688. Basel: EKBB 2019-00816.

Note that full information on the approval of the study protocol must also be provided in the manuscript.

Field-specific reporting

Please select the one below that is the best fit for your research. If you are not sure, read the appropriate sections before making your selection.

☒ Life sciences ☐ Behavioural & social sciences ☐ Ecological, evolutionary & environmental sciences

For a reference copy of the document with all sections, see nature.com/documents/nr-reporting-summary-flat.pdf

Life sciences study design

All studies must disclose on these points even when the disclosure is negative.

Sample size	No power-calculations were performed. The explorative nature of the study did not allow for estimating effect sizes prior to the analysis.
Data exclusions	Data on 16 CITE-seq antibodies, that were not used in all comparisons was excluded from the final version of the manuscript.
Replication	Multiple patients' samples were used for the healthy PBMCs, Immunotherapy treated PBMCs, healthy skin, primary melanoma and metastatic melanoma in the lymph node.
Randomization	The randomization was not relevant for the study as this is a descriptive study describing method establishment.
Blinding	The blinding was not relevant for the study as this is a descriptive study describing method establishment.

Reporting for specific materials, systems and methods

We require information from authors about some types of materials, experimental systems and methods used in many studies. Here, indicate whether each material, system or method listed is relevant to your study. If you are not sure if a list item applies to your research, read the appropriate section before selecting a response.

Materials & experimental systems

n/a	Involved in the study
☐	☒ Antibodies
☒	☐ Eukaryotic cell lines
☒	☐ Palaeontology and archaeology
☐	☒ Animals and other organisms
☐	☒ Clinical data
☒	☐ Dual use research of concern
☒	☐ Plants

Methods

n/a	Involved in the study
☒	☐ ChIP-seq
☐	☒ Flow cytometry
☒	☐ MRI-based neuroimaging

Antibodies

Antibodies used	Oligo conjugated antibodies commercially available from Biolegend. Information for all 97 antibodies used for CITE-seq are listed in supplementary table 1.<br>Antibodies used for mIHC: 1. anti-MelanA, NovusBio, cat.no. NBP1-30151, clone: A19-p; 2. anti-CD68, Abcam, cat.no. ab213363, clone:EPR20545; 3. anti-CD56, Abcam, cat. no. ab220360 ;clone:EPR21827, 4. anti-CD8, Abcam cat.no. ab4055, clone: SP239.<br>Antibodies used for flow cytometry: anti-human CD3 (APC, BioLegend, cat. no. 317318), anti-human CD4 (PerCP, BioLegend, cat. no. 317432), anti-human CD8 (PE-Cy7, BioLegend, cat. no. 344712), anti-human CTLA-4 (Biolegend, clone BNI3, APC/Fire 750, cat. no. 369627) or Mouse IgG2a, κ Isotype control (APC/Fire 750, Biolegend, cat. no. 400283), anti-human CTLA-4 (PE, Biolegend, clone L3D10, cat. no. 349905), PE Mouse IgG1, κ Isotype control (PE, Biolegend, cat. no. 400113).
Validation	For mIHC antibody validation was performed prior multiplex staining, as single staining for a positive tissue recommended by the supplier.

Animals and other research organisms

Policy information about studies involving animals; ARRIVE guidelines recommended for reporting animal research, and Sex and Gender in Research

Laboratory animals	NA
Wild animals	NA
Reporting on sex	NA
Field-collected samples	NA
Ethics oversight	NA

Note that full information on the approval of the study protocol must also be provided in the manuscript.

Clinical data

Policy information about clinical studies

All manuscripts should comply with the ICMJE guidelines for publication of clinical research and a completed CONSORT checklist must be included with all submissions.

Clinical trial registration	NA
Study protocol	NA
Data collection	Limited clinical data is provided in Supplementary Dable 1 with sample origin, patient's/donor's gender and mutational status for melanoma samples.
Outcomes	NA

Flow Cytometry

Plots

Confirm that:

☒ The axis labels state the marker and fluorochrome used (e.g. CD4-FITC).

☒ The axis scales are clearly visible. Include numbers along axes only for bottom left plot of group (a 'group' is an analysis of identical markers).

☒ All plots are contour plots with outliers or pseudocolor plots.

☒ A numerical value for number of cells or percentage (with statistics) is provided.

Methodology

Sample preparation	Live-frozen PBMCs from three patients receiving immunotherapy (Supplementary Table 1) were thawed by drop-wise resuspension in media (RPMI 1640 (Sigma-Aldrich, cat. no. R0883) supplemented with 5 nM L-glutamine (Gibco, Thermo Scientific, cat. no. 25030-024), 1 mM sodium pyruvate (Sigma-Aldrich, cat. no. S8636), 10% heat-inactivated fetal bovine serum (Biowest, cat. no. S181H) and 1% Pen-Strep (Gibco, Thermo Scientific, cat. no. 15140-122)) and rested on ice for 10 minutes. Cells were counted with AOPI (Logos Biosystems, cat. no. F23002) and viability was found to be over 90%. Cells were seeded in round bottom 96 well plates in media at 0.5 Mio cells/well and incubated with or without cell stimulation/protein export inhibitor cocktail (eBioscience, cat. no. 00-4975-93) for 4 hours at 37 °C and 5% CO2. Next, cells were washed with PBS with 2% FCS, and stained with anti-human CD3 (APC, BioLegend, cat. no. 317318), anti-human CD4 (PerCP, BioLegend, cat. no. 317432), anti-human CD8 (PE-Cy7, BioLegend, cat. no. 344712), anti-human CTLA-4 (Biolegend, clone BNI3, APC/Fire 750, cat. no. 369627) or Mouse IgG2a, κ Isotype control (APC/Fire 750, Biolegend, cat. no. 400283), anti-human CTLA-4 (PE, Biolegend, clone L3D10, cat. no. 349905) or PE Mouse IgG1, κ Isotype control (PE, Biolegend, cat. no. 400113). Compensation was performed with UltraComp eBeads™ (Invitrogen, cat. no. 01-2222-42).
Instrument	Samples were analyzed on a LSRFortessa™ Cell Analyzer (BD Biosciences).
Software	The flow cytometry data were analyzed in FlowJo™ v10.0.8.
Cell population abundance	NA
Gating strategy	Cells were gated as lymphocytes/single-cells/CD3+/CD4+CD8- cells on either unstimulated cells (ctrl) or PMA/ionomycin stimulated cells (stim).

☒ Tick this box to confirm that a figure exemplifying the gating strategy is provided in the Supplementary Information.
